# Cyclic di-GMP Signaling Links Biofilm Formation and Mn(II) Oxidation in Pseudomonas resinovorans

**DOI:** 10.1128/mbio.02734-22

**Published:** 2022-11-14

**Authors:** Ainelén Piazza, Lucia Parra, Lucila Ciancio Casalini, Federico Sisti, Julieta Fernández, Jacob G. Malone, Jorgelina Ottado, Diego O. Serra, Natalia Gottig

**Affiliations:** a Instituto de Biología Molecular y Celular de Rosario, Consejo Nacional de Investigaciones Científicas y Técnicas, Universidad Nacional de Rosario, Rosario, Argentina; b Molecular Microbiology Department, John Innes Centre, Norwich, United Kingdom; c Instituto de Biotecnología y Biología Molecular-CCT-CONICET-La Plata, Departamento de Ciencias Biológicas, Facultad de Ciencias Exactas, Universidad Nacional de La Plata, Buenos Aires, Argentina; d School of Biological Sciences, University of East Anglia, Norwich, United Kingdom; Georgia Institute of Technology School of Biological Sciences

**Keywords:** *Pseudomonas*, biofilms, bioremediation, c-di-GMP, metal oxidation

## Abstract

Bioaugmentation of biological sand filters with Mn(II)-oxidizing bacteria (MOB) is used to increase the efficiency of Mn removal from groundwater. While the biofilm-forming ability of MOB is important to achieve optimal Mn filtration, the regulatory link between biofilm formation and Mn(II) oxidation remains unclear. Here, an environmental isolate of Pseudomonas resinovorans strain MOB-513 was used as a model to investigate the role of c-di-GMP, a second messenger crucially involved in the regulation of biofilm formation by Pseudomonas, in the oxidation of Mn(II). A novel role for c-di-GMP in the upregulation of Mn(II) oxidation through induction of the expression of manganese-oxidizing peroxidase enzymes was revealed. MOB-513 macrocolony biofilms showed a strikingly stratified pattern of biogenic Mn oxide (BMnOx) accumulation in a localized top layer. Remarkably, elevated cellular levels of c-di-GMP correlated not only with increased accumulation of BMnOx in the same top layer but also with the appearance of a second BMnOx stratum in the bottom region of macrocolony biofilms, and the expression of *mop* genes correlated with this pattern. Proteomic analysis under Mn(II) conditions revealed changes in the abundance of a PilZ domain protein. Subsequent analyses supported a model in which this protein sensed c-di-GMP and affected a regulatory cascade that ultimately inhibited *mop* gene expression, providing a molecular link between c-di-GMP signaling and Mn(II) oxidation. Finally, we observed that high c-di-GMP levels were correlated with higher lyophilization efficiencies and higher groundwater Mn(II) oxidation capacities of freeze-dried bacterial cells, named lyophiles, showing the biotechnological relevance of understanding the role of c-di-GMP in MOB-513.

## INTRODUCTION

The presence of soluble manganese Mn(II) affects the quality of groundwater, a source of drinking water for many populations, and is an important environmental concern (1–3). Biological sand filter technology, based on bacterial oxidation of metals to form insoluble oxides that can be filtered out of the water, is widely used for groundwater potabilization. Bioaugmentation of this process through the inoculation of sand filters with appropriate Mn(II)-oxidizing bacteria (MOB) optimizes Mn removal (4–8).

Biofilms are sessile and densely populated communities of bacterial cells surrounded by an extracellular matrix that typically contains exopolysaccharides, proteins, or extracellular DNA and serves as a shield that protects the cells from external aggressions and stresses (9). The biofilm-forming capability of bacteria is important to achieve the optimal filtration of groundwater for metals, as they have to be oxidized and retained in the biofilter matrix surface, and to decrease loss of MOBs, which can otherwise be washed out of the system (4, 5, 8). It was recently shown that powdered MOB inoculates prepared by vacuum lyophilization are useful to inoculate sand filters and remove Mn with high efficiencies (10). Moreover, lyophilization efficiencies increase when MOB are grown under static instead of shaking culture conditions (10), demonstrating that an understanding of biofilm formation in MOB may help to implement these bacteria in biotechnological applications.

Previous work with the environmental isolate Pseudomonas resinovorans MOB-513 has shown that the Mn(II) oxidation phenotype in this bacterium is biofilm dependent (11). Accordingly, in Pseudomonas putida GB-1, Mn(II) oxidation was associated with the sessile lifestyle and was influenced by flagella synthesis and by contact with surfaces (12). These results suggest a correlation between an Mn-oxidizing phenotype and biofilm growth.

In Pseudomonas spp., the main regulator of biofilm formation is the second messenger bis-(3′-5′)-cyclic dimeric GMP (c-di-GMP). This ubiquitous signaling molecule controls the transition of bacteria from a motile to a sessile lifestyle and vice versa. In almost all cases, high cellular levels of c-di-GMP promote biofilm formation, while low c-di-GMP levels stimulate bacterial motility and often biofilm dispersal (13). The cellular pool of c-di-GMP is regulated by GGDEF domain-containing diguanylate cyclases (DGCs), which synthesize the messenger, and EAL/HD-GYP-domain-containing phosphodiesterases (PDEs), which degrade it (13). c-di-GMP binds to an array of intracellular receptors (RNA riboswitches, PilZ domains, degenerate GGDEF/EAL domains, and numerous other protein folds) that go on to exert specific cellular effects at transcriptional, translational, and posttranslational levels (14, 15).

The bacterial enzymes involved in Mn(II) oxidation (Mn oxidases) that have been characterized so far belong to two families of proteins: the multicopper oxidases (MCOs) and manganese peroxidases (MOPs) (16). P. putida GB-1 has two MCO-type enzymes with Mn oxidase activities, MnxG and McoA (17). The expression of *mnxG* and *mcoA* genes is positively regulated by a two-component regulatory pathway, although the external signals inducing the system remain unclear (18). The MOP enzyme MopA, implicated in directly oxidizing Mn(II), was also found in P. putida GB-1 (19). In this bacterium, the deletion of the transcriptional regulator *fleQ* results in the overproduction and secretion of MopA, while the activities of both MnxG and McoA decrease (19). FleQ is a c-di-GMP-responsive transcription factor, which binds c-di-GMP as a function of the cellular levels of this messenger, to regulate flagella synthesis and biofilm formation (20). This not only suggests that different Mn(II) oxidases function in planktonic and biofilm cells (19) but also implies a connection between c-di-GMP signaling and the Mn(II) oxidation process.

Collectively, this led us to hypothesize that c-di-GMP and its associated signaling network is the regulatory link between Mn(II) oxidation and biofilm formation in Pseudomonas. Therefore, MOB-513 was used as a bacterial model to investigate for the first time how variations in c-di-GMP levels—achieved through the ectopic expression of DGCs or PDEs—influence Mn(II) oxidation. Specifically, we determined the effect of c-di-GMP levels on Mn(II) oxidation phenotypes in MOB-513, demonstrating a novel role for this messenger in the upregulation of this process and highlighting the feasibility of generating hyperoxidizer bacterial strains to optimize Mn removal from groundwater.

## RESULTS

### Analysis of the MOB-513 genome revealed several genes potentially involved in Mn(II) oxidation and c-di-GMP metabolism.

The complete MOB-513 genome sequence was analyzed (see Fig. S1 in the supplemental material) to gain better insights into the mechanism of Mn(II) oxidation and c-di-GMP metabolism. We identified potential Mn(II) oxidases by comparison to experimentally verified MnxG, McoA, and MopA enzymes from P. putida GB-1 (17, 19). This analysis revealed two genes, *Pres513_3924* and *Pres513_3296*, homologous to *mnxG* and *mcoA*, respectively. Like P. putida GB-1 MnxG and McoA proteins, *Pres513_3924* and *Pres513_3296* encode proteins with a conserved multicopper oxidase domain (Table 1). Moreover, three genes homologous to *mopA* were detected: *Pres513_7013*, *Pres513_7014*, and *Pres513_5806*. Like P. putida GB-1 MopA, these genes encode proteins with two animal heme peroxidase conserved domains and several hemolysin-type calcium-binding domains (Table 1).

**TABLE 1:** Genes in strain MOB-513 with sequence homology to putative Mn(II) oxidases from P. putida GB-1

Locus tag in strain		Identity	Similarity	E value	Conserved domain(s) found in proteins^*a*^
P. putida GB-1	MOB-513				
*PputGB1_2447* (*mnxG*)	*Pres513_3924* (*mco3924*)	74%	83%	0.0	Multicopper oxidase (PS00080)
*PputGB1_2665* (*mcoA*)	*Pres513_3296* (*mco3296*)	31%	45%	2e−76	Multicopper oxidase (PS00080)
*PputGB1_3353* (*mopA*)	*Pres513_7013* (*mop7013*)	55%	66%	2e−11	Animal heme peroxidase (PS50292), hemolysin-type calcium-binding (PS00330)
*PputGB1_3353* (*mopA*)	*Pres513_7014* (*mop7014*)	52%	63%	3e−20	Animal heme peroxidase (PS50292), hemolysin-type calcium-binding (PS00330)
*PputGB1_3353* (*mopA*)	*Pres513_5806* (*mop5806*)	65%	74%	3e−13	Animal heme peroxidase (PS50292), hemolysin-type calcium-binding (PS00330)

a Predicted by a PROSITE domain scan (https://prosite.expasy.org/) (48). Accession numbers are shown in parentheses.

10.1128/mbio.02734-22.1 FIG S1 Subsystem classification of *P. resinovorans* strain MOB-513 genome properties by RAST annotation (https://rast.nmpdr.org/). The 7,489,538-bp genome of MOB-513 was sequenced and had a GC content of 62.5%, with 7,209 coding sequences. RAST annotation allowed the classification in subsystems. The most abundant categories were related to maintaining basal cell functions: Amino acids and derivatives metabolism; Carbohydrates metabolism; Cofactors, vitamins, prosthetic groups; Fatty acids, lipids, and isoprenoids metabolism; Protein metabolism; Aromatic compounds metabolism (that is a characteristic prominent metabolism in environmental Pseudomonas); DNA and RNA metabolism; membrane Transport; Stress response. Download FIG S1, TIF file, 0.4 MB. Copyright © 2022 Piazza et al. 2022 Piazza et al. https://creativecommons.org/licenses/by/4.0/ This content is distributed under the terms of the Creative Commons Attribution 4.0 International license.

Additionally, we identified 21 proteins containing the GGDEF domain, i.e., putative DGCs, and 14 proteins containing both GGDEF and EAL domains.

### Overexpression of DgcB or PdeA in MOB-513 changed intracellular c-di-GMP levels, inversely regulating biofilm formation and swimming motility.

**FIG 1:**
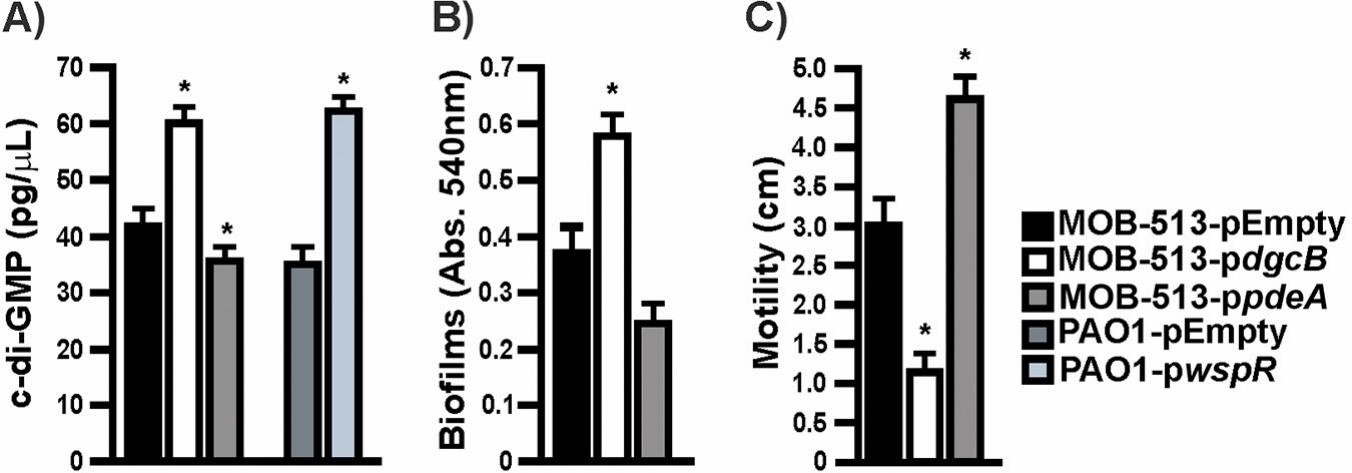
c-di-GMP levels, biofilm formation, and swimming motility in MOB-513 strains overexpressing DgcB or PdeA. (A) c-di-GMP concentration in cells of *P. resinovorans* MOB-513 and P. aeruginosa PAO1 strains. c-di-GMP levels were quantified using the c-di-GMP assay kit (Lucerna). P. aeruginosa strains PAO1-p*WspR* and PAO1-pEmpty were used as controls for the assays. (B) Biofilm formation by MOB-513 strains at the air-liquid interface assessed by the CV assay. (C) Migration zone diameters of the MOB-513 strains grown on LB swimming plates for 72 h at 28°C. Quantifications were performed in triplicate, and mean values ± SD are presented. Data were statistically analyzed using a one-way ANOVA, and asterisks indicate significant differences compared to the controls (*P* < 0.05).

The large number of genes encoding putative DGCs and/or PDEs found in MOB-513 suggested that a complex c-di-GMP signaling network operates in this strain, making it difficult to predict which of these enzymes act to globally regulate the cellular pool of c-di-GMP. Thus, to increase and decrease the intracellular levels of c-di-GMP in MOB-513, we opted to ectopically overexpress *dgcB* and *pdeA*, well-characterized DGC and PDE genes from Bordetella bronchiseptica, respectively (21, 22). MOB-513 overexpressing DgcB (MOB-513-p*dgcB*) presented significantly higher c-di-GMP levels than the MOB-513 strain harboring the pEmpty vector (MOB-513-pEmpty); inversely, MOB-513 overexpressing PdeA (MOB-513-p*pdeA*) showed significantly lower c-di-GMP levels than MOB-513-pEmpty (Fig. 1A). These results were additionally supported by measurements of [c-di-GMP] in P. aeruginosa PAO1 overexpressing the DGC gene *wspR*, which was previously shown to overproduce c-di-GMP, and in the corresponding control strain (23) (Fig. 1A).

To corroborate the physiological effects of raising and lowering c-di-GMP levels in MOB-513, biofilm formation and motility, two bacterial phenotypes shown to be inversely regulated by c-di-GMP in other bacteria (13), were analyzed. As expected, MOB-513-p*dgcB* showed an ~1.5-fold increase in biofilm biomass accumulation and a 2.8-fold decrease in swimming motility relative to MOB-513-pEmpty (Fig. 1B and C). Conversely, in MOB-513-p*pdeA*, a 1.4-fold reduction in biofilm biomass and an ~1.5-fold increase in swimming motility compared to the control strain was observed (Fig. 1B and C).

### Changes in c-di-GMP levels strongly influenced the onset and performance of Mn(II) oxidation by MOB-513 in biofilms.

**FIG 2:**
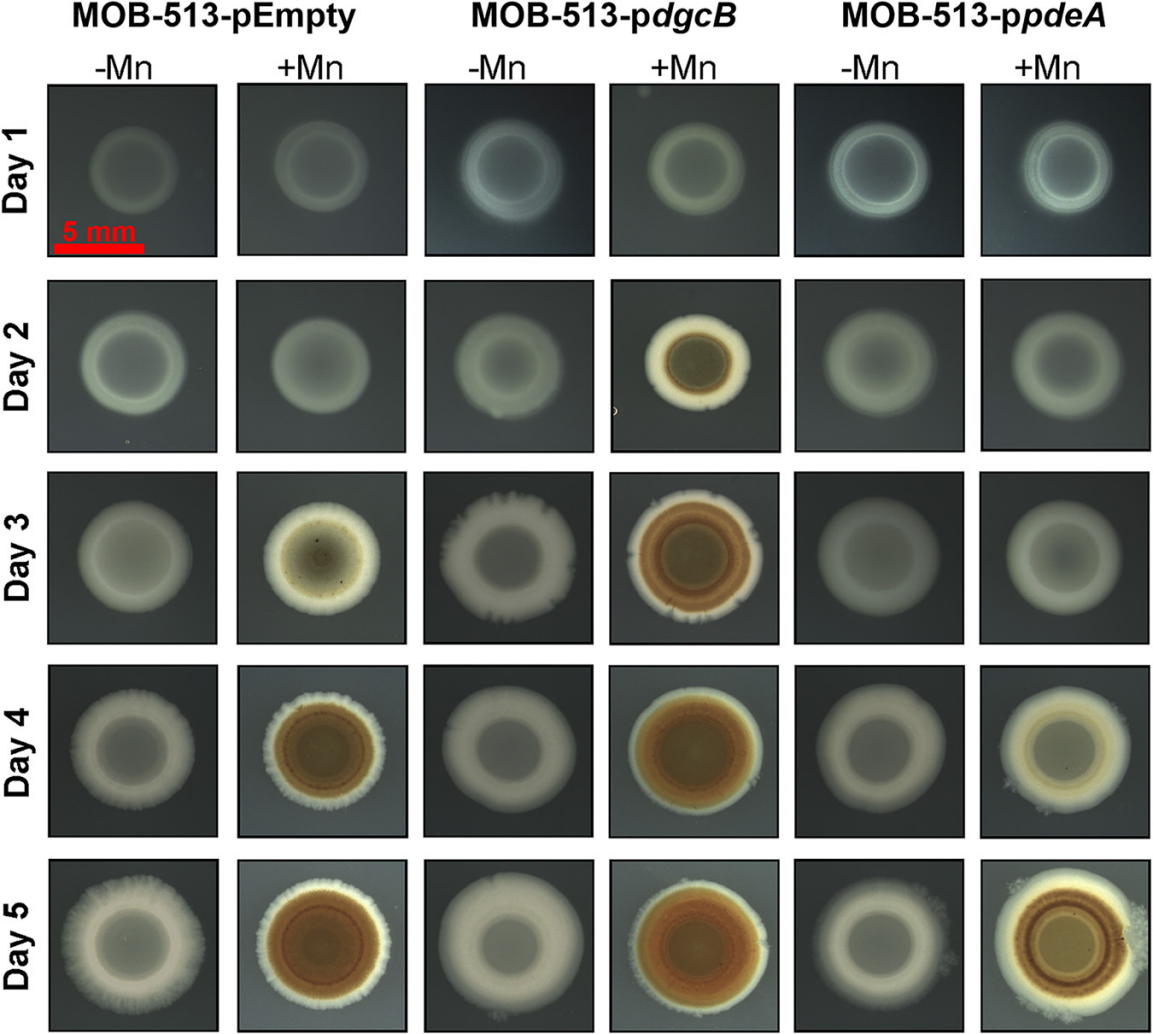
Time course of Mn(II) oxidation in macrocolony biofilms of MOB-513 strains. Top view shows MOB-513-pEmpty, MOB-513-p*dgcB*, and MOB-513-p*pdeA* macrocolonies at distinct stages of growth, exhibiting (or not) Mn(II) oxidation phenotypes (brown color). Macrocolonies were set on Lept (−Mn) and Lept-Mn (+Mn) plates, incubated at 28°C and imaged daily for 5 days. Red scale bar, 5 mm.

In large colony biofilms, known as macrocolonies (24), the formation of biogenic Mn oxide (BMnOx) by MOBs can be readily visualized by the appearance of brown color when these biofilms are grown on Lept agar medium supplemented with Mn(II) (11). Thus, we used this biofilm model system to evaluate the effect of increasing or decreasing the cellular levels of c-di-GMP on the Mn(II) oxidation performance of MOB-513. As shown in Fig. 2A and consistent with previous observations (11), in the presence of Mn(II), macrocolonies of the MOB-513 control strain started to develop light brown coloration by day 3, which became much more intense and visible by days 4 and 5, when the biofilms reached their maximum expansion. This phenotype was strictly dependent on the presence of Mn(II) in the medium. Remarkably, in macrocolonies of MOB-513-p*dgcB*, the brown color appeared as early as day 2 (Fig. 2), indicating that increased c-di-GMP levels accelerated the onset of BMnOx formation. Conversely, in macrocolonies of MOB-513-p*pdeA*, the appearance of BMnOx was delayed, starting at day 4 and becoming readily visible by day 5 (Fig. 2). This opposite effect reinforced the involvement of c-di-GMP in regulating the onset of Mn(II) oxidation by MOB-513.

**FIG 3:**
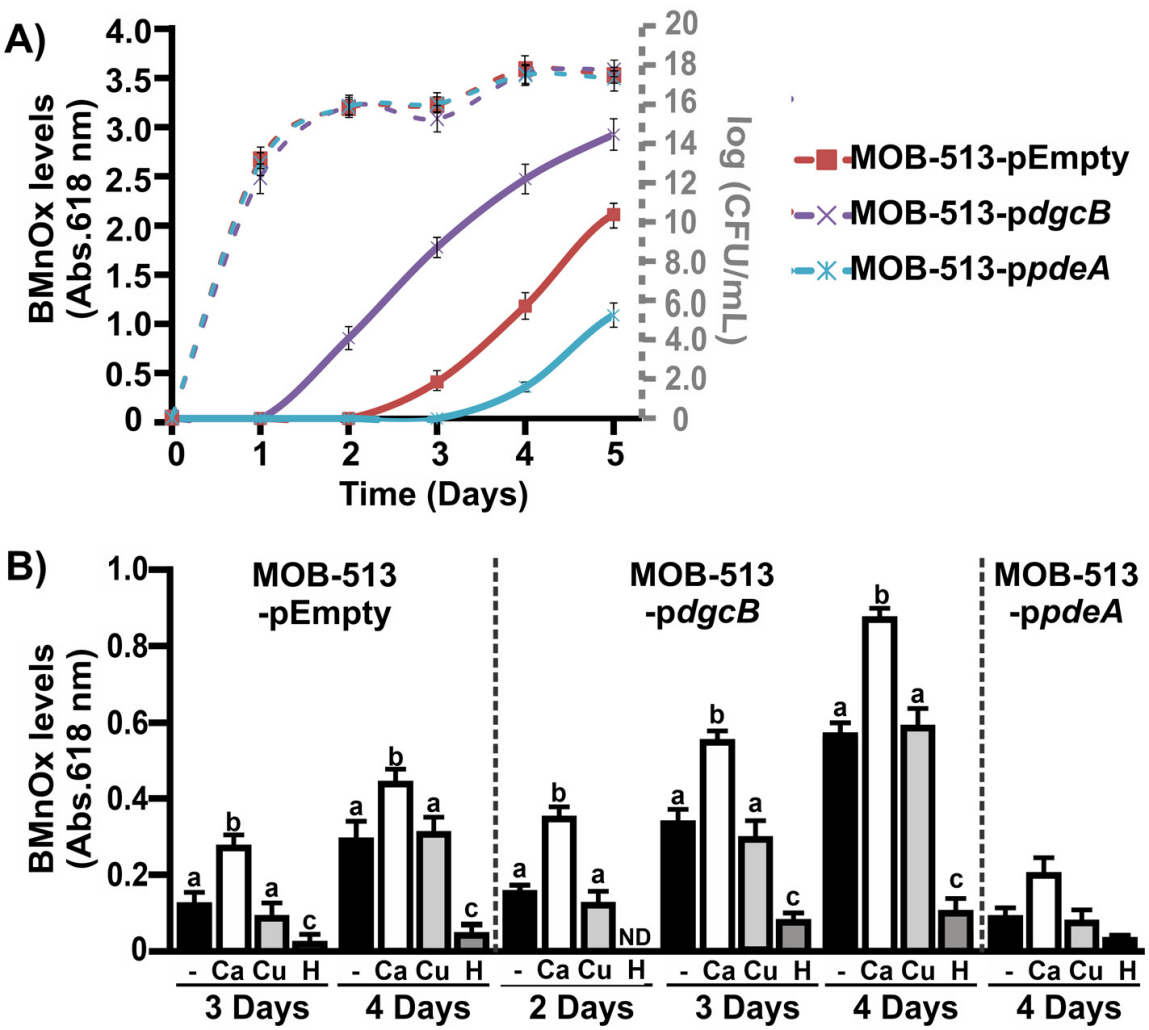
Involvement of c-di-GMP signaling in Mn(II) oxidation by MOB-513 strains. (A) Mn(II) oxidation capacities of MOB-513-pEmpty, MOB-513-p*dgcB*, and MOB-513-p*pdeA* strains. Quantification of BMnOx formed by the strains was assessed daily for 5 days using the LBB assay. Axis on the left side presents values of absorbance at 618 nm as determination of BMnOx (solid lines). Axis on the right side of the plot (gray color) presents log CFU per milliliter values as determination of growth (dashed lines). Absorbance and log CFU per milliliter values represent the mean of values from three biological replicates at each time point analyzed. Error bars indicate the SD. Data were statistically analyzed using one-way ANOVA (*P* < 0.05). (B) *In vitro* Mn(II) oxidase activities, assayed in total protein extracts obtained from MOB-513-pEmpty, MOB-513-p*dgcB*, and MOB-513-*pdeA* macrocolonies grown on Lept-Mn for 2, 3, and 4 days. Quantifications were performed from three biological replicates. Mean values and SD are presented. Data were statistically analyzed using one-way ANOVA followed by Tukey’s test. Bars with different letters (a, b, c, and d) indicate significant differences between treatments (*P* < 0.05). ND, no activity was detected.

Quantitative analysis of BMnOx accumulation and Mn oxidase activity assays over time in macrocolony biofilms of the three MOB-513 strains were performed using the leucoberbelin blue (LBB) assay. Elevated cellular levels of c-di-GMP correlated with early and increased BMnOx production in macrocolonies (~1.6-fold higher than the amount of BMnOx produced by the control MOB-513-pEmpty strain at day 5), while decreased cellular levels of c-di-GMP led to delayed and diminished BMnOx production (about half of the BMnOx yielded by the control MOB-513-pEmpty strain at day 5). These effects were related specifically to changes in the cellular levels of c-di-GMP, as macrocolonies of the three MOB-513 strains showed essentially the same growth pattern, as determined by viable cell counting (Fig. 3A).

Mn oxidase activities correlated with BMnOx production and no evidence of Mn oxidase activity in heat-treated control samples was detected (*P* < 0.05), demonstrating that Mn(II) oxidation occurs in MOB-513 through enzymatic processes (Fig. 3B). Moreover, two metal ions, Ca(II) and Cu(II), were tested for their effects on enzymatic activity, and they enhanced MopA (25) and MCO activities (26), respectively. Only the presence of Ca(II) significantly enhanced the Mn(II)-oxidizing activities (Fig. 3B), suggesting the involvement of MOPs in the Mn(II) oxidation process in MOB-513.

Altogether, these results not only confirmed that changes in the cellular levels of c-di-GMP altered the onset of Mn(II) oxidation but also showed that such changes strongly influenced the overall yield of BMnOx produced by inducing shifts in enzymatic activity.

### BMnOx accumulation occurred in well-defined and restricted zones of MOB-513 biofilms in a c-di-GMP-dependent manner.

**FIG 4:**
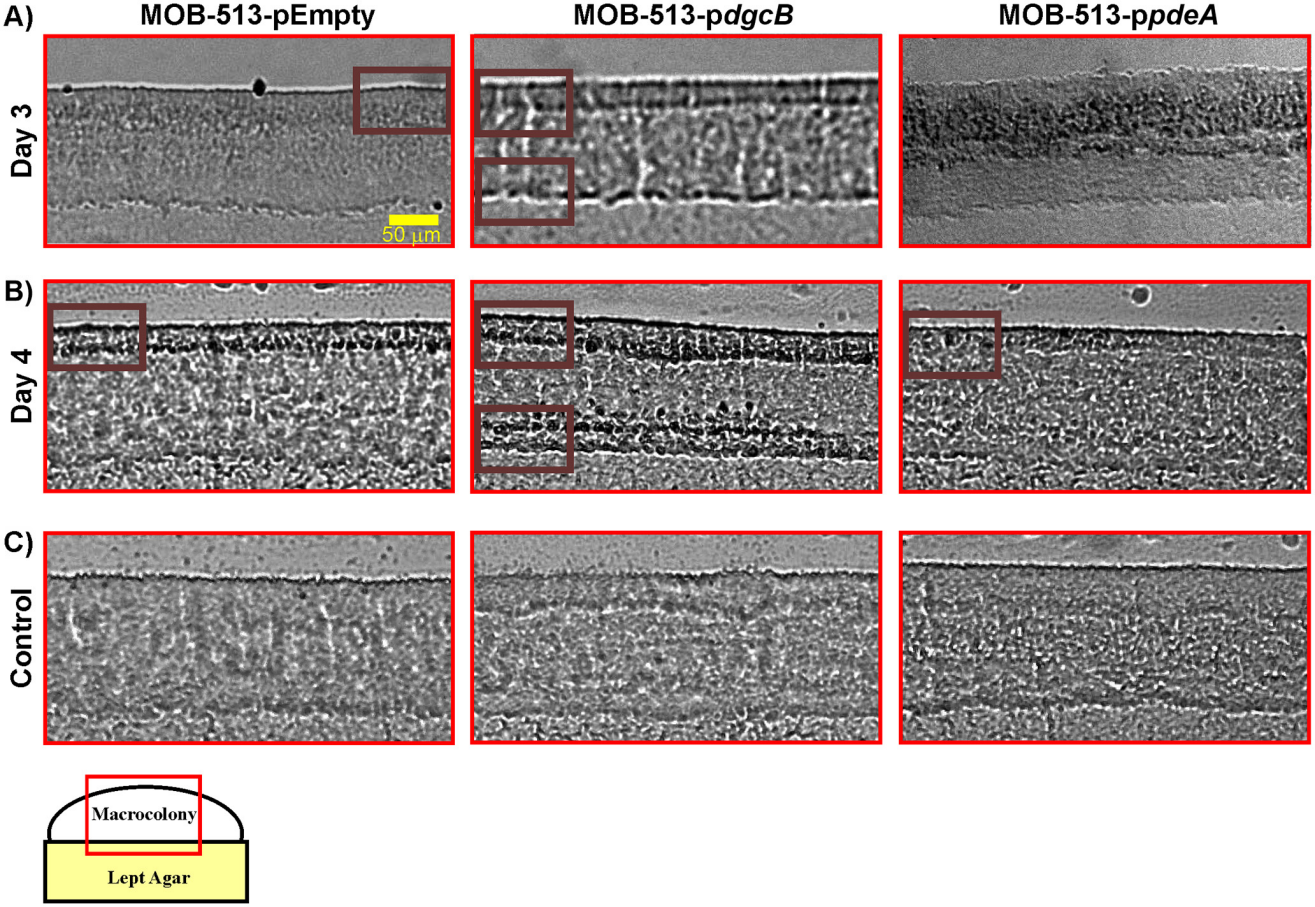
Cryosectioning and bright-field microscopy approach to analyze BMnOx depositions. (A and B) Images of 5-μm-thin vertical sections of 3- and 4-day-old macrocolonies, respectively, of MOB-513-pEmpty, MOB-513-p*dgcB*, and MOB-513-p*pdeA* strains grown on Lept-Mn medium. Dark areas (Bordeaux boxed) in the images correspond to BMnOx deposited in the top layer of the macrocolonies for all the strains and a second layer of BMnOx located at the bottom of the macrocolony in MOB-513-p*dgcB*. (C) Images of 5-μm-thin vertical sections of 4-day-old macrocolonies of all strains grown in the absence of Mn(II) in the Lept medium. Representative images of phenotypes at day 4 are shown here, but the same results were observed for all the times assayed.

While in macrocolony biofilms brown coloration visually denotes the presence of BMnOx, it does not precisely reveal how the oxidized mineral distributes across the internal section of the biofilm. To gain more insights into this aspect, macrocolony biofilms of MOB-513 strains grown on Lept or Lept-Mn were cross-sectioned and microscopically examined for the presence of BMnOx. In MOB-513-pEmpty, BMnOx started to be detected by day 3 in a localized manner in the upper part of the biofilm (Fig. 4A). Interestingly, by day 4 the amount of BMnOx increased and had strikingly accumulated as a well-defined dark band near the macrocolony surface (Fig. 4B). The same spatial pattern of BMnOx distribution was observed in cross-sections of macrocolonies of MOB-513-p*dgcB*, but at day 3, i.e., 1 day earlier (Fig. 4A). Remarkably, at day 4, MOB-513-p*dgcB* macrocolonies exhibited further accumulation of BMnOx in a second layer at the bottom of the biofilm, close to the nutrient-providing agar and remote from the colony interface with the air (Fig. 4B). In contrast, at day 4 macrocolonies of MOB-513-p*pdeA* showed very little BMnOx in the upper part of the biofilm section (Fig. 4B). The second band of BMnOx found in MOB-513-p*dgcB* macrocolonies was not observed in MOB-513-pEmpty or MOB-513-pB*p*pdeA macrocolonies at any time point assayed. This explains the larger amount of BMnOx detected in MOB-513-p*dgcB* macrocolonies in the LBB assay, compared with macrocolonies of the two other strains. As expected, no accumulation of BMnOx was observed when macrocolonies of the three strains were grown in the absence of Mn(II) (Fig. 4C).

### Expression of MOPs was induced by Mn(II) and c-di-GMP in MOB-513 macrocolonies.

**FIG 5:**
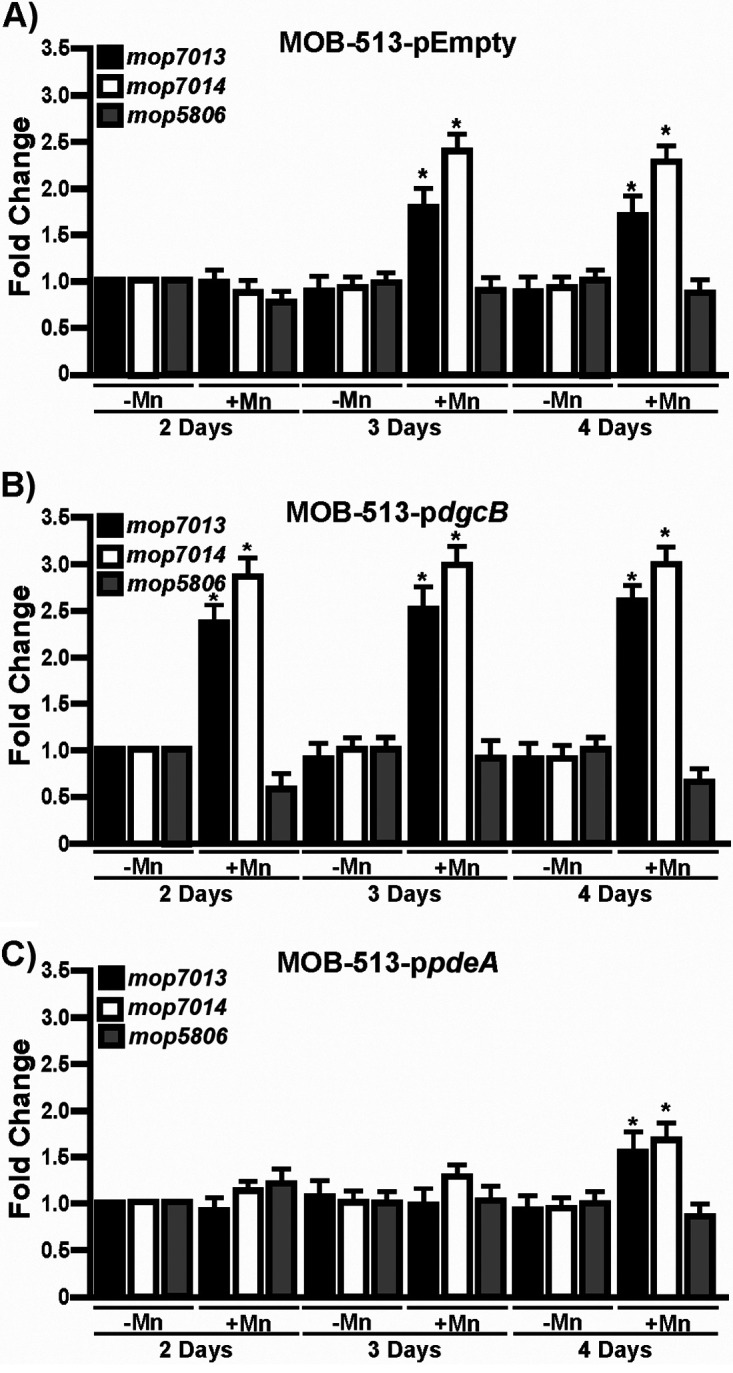
Expression of *mop* genes in MOB-513 strains. Macrocolonies of the strains MOB-513-pEmpty (A), MOB-513-p*dgcB* (B), and MOB-513-p*pdeA* (C) were grown on Lept and Lept-Mn and subjected to qRT-PCR assays to analyze the expression of *mop7013*, *mop7014*, and *mop5806*. The *rpoD* gene was used as internal control for the calculation of relative gene expression. Bars indicate the expression levels of the genes in Lept-Mn relative to the expression levels in the absence of Mn(II). Values are the means of three biological replicates. Error bars indicate standard deviations. Data were analyzed by Student's *t* test, and asterisks indicate significant differences (*P* < 0.05) between samples grown in the presence and absence of Mn(II).

**FIG 6:**
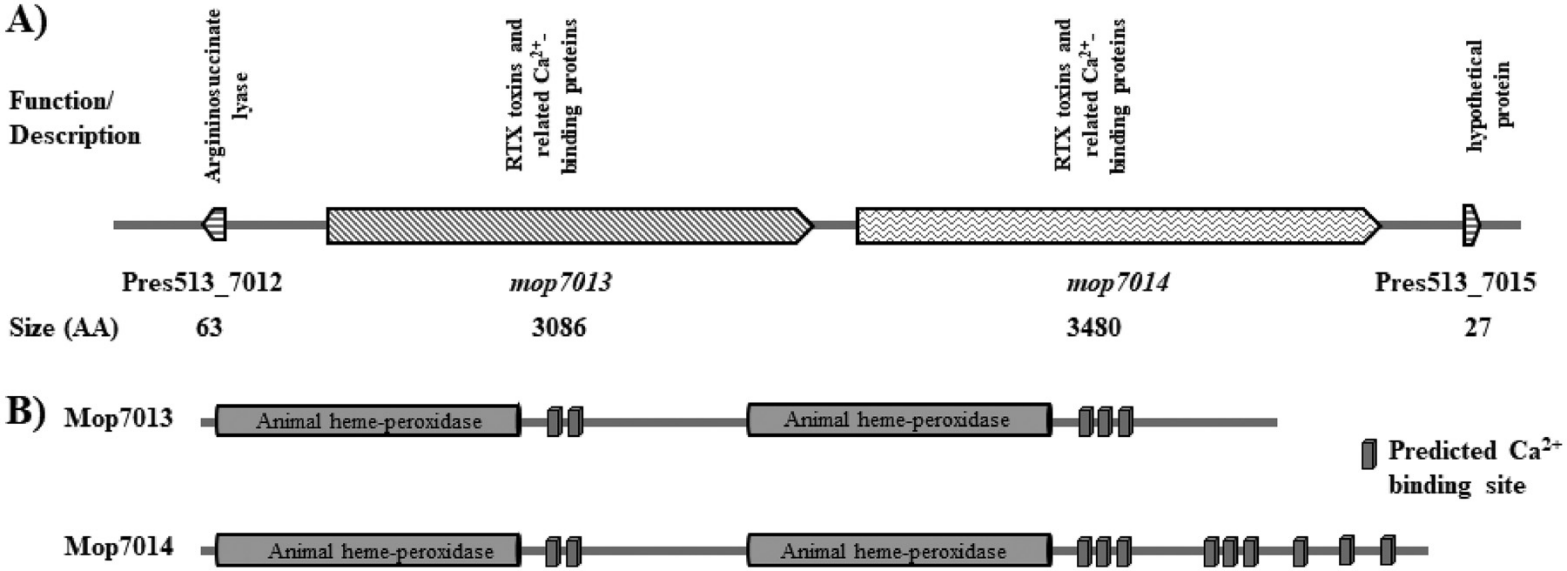
(A) The MOB-513 chromosomal region centered on the focused genes *mop7013* and *mop7014* from *P. resinovorans* strain MOB-513. The online software Softberry was used for prediction of *mop7013* and *mop7014* promoter regions. The figure shows each open reading frame as an arrow filled with different patterns and the sizes of the proteins as the number of amino acids. Arrows pointing to the right indicate that genes are on the forward strand, and arrows to the left indicate that they are on the reverse one. Predicted functions and descriptions have been obtained from the RAST Server (https://rast.nmpdr.org/). (B) Mop7013 and Mop7014 from Pseudomonas resinovorans strain MOB-513 with predicted animal heme peroxidase domains and Ca(II) binding regions labeled. Both Mop7013 and Mop7014 contained two peroxidase domains related to the animal heme peroxidase superfamily (PROSITE accession number PS50292), and following each of these domains were 5 and 11 hemolysin-type Ca(II) binding regions (PROSITE accession number PS00330), respectively.

To understand which Mn(II) oxidases are activated in the different MOB-513 macrocolony biofilms, the transcriptional profiles of *mco3924*, *mco3296*, *mop7013*, *mop7014*, and *mop5806* genes (Table 1) were analyzed. The expression levels of *mco3924*, *mco3296*, and *mop5806* were unaffected under biofilm conditions in any strain in the presence of Mn(II) at any of the assayed times (Fig. S2 and Fig. 5A to C). However, *mop7013* and *mop7014* were differentially expressed. In MOB-513-pEmpty, both genes showed increased expression in the presence of Mn(II) from day 3 (*P* < 0.05) (Fig. 5A). In MOB-513-p*dgcB*, *mop7013* and *mop7014* mRNA abundance was induced earlier (day 2) and increased relative to MOB-513-pEmpty (*P* < 0.05) (Fig. 5B). In MOB-513-p*pdeA*, a reduced and delayed induction of both genes compared with MOB-513-pEmpty was observed (*P* < 0.05) (Fig. 5C). The timing of *mop7013* and *mop7014* gene expression correlated with the appearance of BMnOx and Mn oxidase activity in the tested strains (Fig. 2 and 3), suggesting that Mop7013 and Mop7014 are involved in Mn(II) oxidation in MOB-513 macrocolony biofilms. These two predicted peroxidases shared a conserved domain architecture with the characterized MopA proteins from Aurantimonas manganoxydans strain SI85-9A1 and P. putida GB-1 (19, 25) (Fig. 6).

10.1128/mbio.02734-22.2 FIG S2 Analysis of *mco* gene expression levels in the different strains. Macrocolonies of strains MOB-513-pEmpty (A), MOB-513-p*dgcB* (B), and MOB-513-p*pdeA* (C) were grown on Lept and Lept-Mn and subjected to qRT-PCR assays (as described in Fig. 5) to analyze *mco3924* and mco*3296* gene expression. Bars indicate the expression levels of the genes in Lept-Mn (+Mn) relative to the expression levels at 2 days in the absence of Mn (−Mn). Values are the means of three biological replicates. Error bars indicate standard deviations. Data were analyzed by Student's *t* test, and asterisks indicate significant difference (*P* < 0.05) between samples grown in the presence of Mn(II) and absence of this metal. Download FIG S2, TIF file, 0.1 MB. Copyright © 2022 Piazza et al. 2022 Piazza et al. https://creativecommons.org/licenses/by/4.0/ This content is distributed under the terms of the Creative Commons Attribution 4.0 International license.

### *mop* gene expression spatially correlated with BMnOx production in MOB-513-p*dgcB* macrocolonies.

Macrocolony biofilms represent a highly structured type of biofilm with a clear stratification of metabolic activities (27, 28). In the MOB-513-p*dgcB* macrocolony biofilm, the upper BMnOx layer is further away from the nutrient-providing agar and exposed to a higher oxygen concentration than the bottom BMnOx layer, which is right above the agar surface. Therefore, Mn(II) oxidation may be mediated by different enzymatic activities in each BMnOx-producing cell layer. To study if *mop7013* and *mop7014* are actively expressed in these specific cell layers, *gfp* reporter fusions to their predicted promoters (Fig. 6A) were constructed to analyze their spatial expression patterns in MOB-513-pEmpty and MOB-513-p*dgcB* macrocolony biofilms. Macrocolonies of MOB-513-pEmpty and MOB-513-p*dgcB* transformed with these reporter fusions or the empty pPROBE-KT vector (control) (Table 2) were grown on Lept and Lept-Mn. Green fluorescent protein (GFP) fluorescence was detected in MOB-513-p*dgcB*-p*7013p* and MOB-513-pEmpty-p*7013p*, while no fluorescence was detected for MOB-513-p*dgcB*-p*7014p* or MOB-513-pEmpty-p*7014p* (Fig. S3). In addition, *gfp* expression was induced in the presence of Mn(II) in both MOB-513-pEmpty-p*7013p* and MOB-513-p*dgcB*-p*7013p*, with fluorescence in the latter being specifically enhanced due to the overexpression of DgcB (Fig. S3). Taken together, these results suggest that *mop7013* and *mop7014* expression depends on the promoter found upstream of *mop7013* and that the putative promoter predicted for *mop7014* may not be functional.

**TABLE 2:** Plasmids and strains used in this study

Strain or plasmid	Genotype	Antibiotic	Reference
Plasmids			
pEmpty	pBBR1MCS-5 (with *nptII* promoter)	Gm	(38)
p*dgcB*	pBBR1MCS-5 derived plasmid with *dgcB* inserted downstream the *nptII* promoter	Gm	(21)
p*pdeA*	pBBR1MCS-5 derived plasmid with *pdeA* inserted downstream the *nptII* promoter	Gm	(22)
pPROBE-KT	rep^p^ BBR1promoterless *gfp*	Km	(40)
p*7013p*	pPROBE-KT derived plasmid with the *mop7013* promoter inserted upstream the *gfp* gene	Km	This work
p*7014p*	pPROBE-KT derived plasmid with the *mop7014* promoter inserted upstream the *gfp* gene	Km	This work
p*WspR*19	P. aeruginosa PAO1 containing pBBR2-MCS5 with *wspR*19	Gm	(23)
Strains			
*P. resinovorans* strains			
MOB-513	Manganese oxidizer, wild type	Cm	(11)
MOB-513-pEmpty	*MOB-513* containing pEmpty	Cm, Gm	This work
MOB-513-p*dgcB*	*MOB-513* containing p*dgcB*	Cm, Gm	This work
MOB-513-p*pdeA*	*MOB-513* containing p*pdeA*	Cm, Gm	This work
MOB-513-p*dgcB*-pPROBE-KT	MOB-513-p*dgcB* containing pPROBE-KT	Gm, Km	This work
MOB-513-p*dgcB*-p*7013p*	MOB-513-p*dgcB* containing p*7013p*	Gm, Km	This work
MOB-513-p*dgcB*-p*7014p*	MOB-513-p*dgcB* containing p*7014p*	Gm, Km	This work
MOB-513-pEmpty-pPROBE-KT	MOB-513-pEmpty containing pPROBE-KT	Gm, Km	This work
MOB-513-pEmpty-p*7013p*	MOB-513-pEmpty containing p*7013p*	Gm, Km	This work
MOB-513-pEmpty-p*7014p*	MOB-513-pEmpty containing p*7014p*	Gm, Km	This work
MOB-513-*pilZp*::Tn-pEmpty	MOB-513 with the ALMAR3 transposon inserted into *pilZp* containing pEmpty	Gm, Tc	This work
MOB-513-*pilZp*::Tn-p*dgcB*	MOB-513-pEmpty with the ALMAR3 transposon inserted into *pilZp* containing p*dgcB*	Gm, Tc	This work
P. aeruginosa strains			
PAO1-pEmpty	PAO1 containing pBBR2-MCS5-Empty	Gm	(23)
PAO1-p*WspR*	PAO1 containing p*WspR*19	Gm	(23)

10.1128/mbio.02734-22.3 FIG S3 Fluorescence quantification. Macrocolonies of the strains *dgcB*MOB-513-pEmpty-p*7013p*, MOB-513-pEmpty-p*7014p*, MOB-513-p*dgcB*-p*7013p*, and MOB-513-p*dgcB*-p*7014p* were grown on Lept (−Mn) and Lept-Mn (+Mn), and GFP fluorescence was quantified relative to GFP of the MOB-513-pEmpty-pPROBE-KT or MOB-513-p*dgcB*-pPROBE-KT strains. Quantifications were performed from three biological replicates (expressed in arbitrary units [a.u.]), and SD are presented in the figure. *, significant changes (*P* < 0.05). Download FIG S3, TIF file, 0.1 MB. Copyright © 2022 Piazza et al. 2022 Piazza et al. https://creativecommons.org/licenses/by/4.0/ This content is distributed under the terms of the Creative Commons Attribution 4.0 International license.

**FIG 7:**
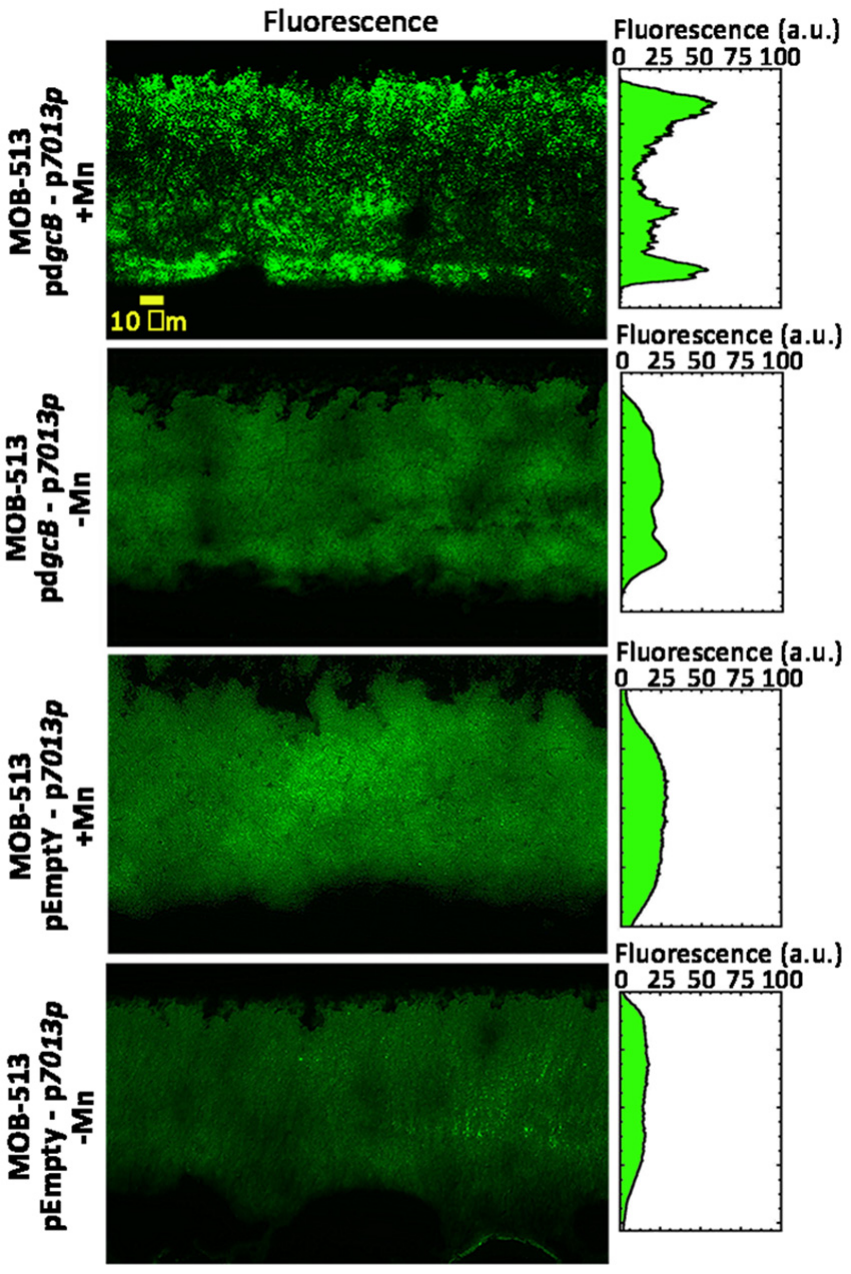
Analysis of spatial expression of the *mop7013* gene in MOB-513-pEmpty and MOB-513-p*dgcB* macrocolony biofilms in the presence and absence of Mn(II). Macrocolonies were grown on Lept (−Mn) and Lept-Mn (+Mn) and thin sectioned. Representative images of fluorescence micrographs, which showed the central region of cross-sections of 3-day-old macrocolonies, are shown. The spectral plot shows fluorescence of the *7013p*::*gfp* reporter fusions as a function of depth across the macrocolony cross-section. Quantification of the spatial distribution of GFP activities of reporter fusions across macrocolony cross-sections was performed using FIJI software. For each reporter fusion or growth condition, the highest fluorescence intensity value in the respective spectrum was arbitrarily set to 100 (in arbitrary units [a.u.]).

Next, 3-day-old macrocolonies of the strains were thin sectioned, and the resulting sections were examined with fluorescence microscopy (Fig. 7). In agreement with our GFP fluorescence quantifications (Fig. S3), cross-sections of the MOB-513-p*dgcB*-p*7014p* and MOB-513-pEmpty-p*7014p* strains did not show fluorescence signals. In the absence of Mn(II), MOB-513-p*dgcB*-p*7013p* showed moderate GFP fluorescence homogenously distributed across the entire section of the macrocolony (Fig. 7). In the presence of Mn(II), a significant increase of GFP expression was observed, with the strongest fluorescence signal occurring in the upper and bottom cell layers (Fig. 7), which correlated with the spatial pattern of BMnOx produced by the strain. In MOB-513-pEmpty-p*7013p* macrocolony cross-sections, the GFP fluorescence was also shown to increase in the presence of Mn(II), but its intensity was lower than the fluorescence observed for MOB-513-p*dgcB*-p*7013p* under the same condition and relatively more homogenous throughout the macrocolony section (Fig. 7).

### High levels of c-di-GMP induced specific changes in the MOB-513 proteome.

To more closely examine how the overexpression of DgcB accelerates and enhances Mn(II) oxidation and to identify proteins besides MopA involved in the MOB-513 Mn(II) oxidation process, label-free quantification was conducted on proteomes from 2-day-old macrocolonies of MOB-513-pEmpty and MOB-513-p*dgcB* (see Fig. S4, Table S1, S2, and S3 and Text S1 in the supplemental material).

10.1128/mbio.02734-22.4 FIG S4 Correlation between the oxidation of Mn(II) and c-di-GMP levels in macrocolonies of MOB-513. (Left) Representative images of 2-day-old macrocolonies of MOB-513-pEmpty and MOB-513-p*dgcB* grown on Lept (−Mn) or Lept-Mn (+Mn) and the heatmap generated from proteomic data obtained for the respective macrocolonies. Rows and columns represent independent samples (3 biological replicates for each condition) and proteins, respectively. Red indicates higher protein abundance, while blue indicates lower protein abundance. The dendrogram represents results from hierarchical clustering and depicts similarities (Pearson correlation) between protein levels under the tested conditions. Cluster 1 contains 62 proteins that were downregulated with the overexpression of DgcB, while cluster 2 contains 60 proteins that were upregulated with the overexpression of DgcB (see Table S1). Download FIG S4, TIF file, 1.0 MB. Copyright © 2022 Piazza et al. 2022 Piazza et al. https://creativecommons.org/licenses/by/4.0/ This content is distributed under the terms of the Creative Commons Attribution 4.0 International license.

10.1128/mbio.02734-22.8 TEXT S1 Proteomic Supplementary Information. Supplementary information of proteome analyses. Download Text S1, PDF file, 0.1 MB. Copyright © 2022 Piazza et al. 2022 Piazza et al. https://creativecommons.org/licenses/by/4.0/ This content is distributed under the terms of the Creative Commons Attribution 4.0 International license.

Both Mop7013 and Mop7014 were detected only in strain MOB-513-p*dgcB* (Table S2 and S3), consistent with the higher expression levels of their genes in this strain compared to MOB-513-pEmpty. Interestingly, for MOB-513-pEmpty, a GGDEF/EAL domain protein (Pres513_4541) showed increased levels in the presence of Mn(II) (Table S1). Furthermore, for MOB-513-p*dgcB*, a hypothetical PilZ domain protein (Pres513_6471), was Mn(II) upregulated (Table S1).

10.1128/mbio.02734-22.5 TABLE S1 KEGG pathway analysis of down- and upregulated proteins in MOB-513-pEmpty and MOB-513-p*dgcB* in the presence of Mn(II). Download Table S1, PDF file, 0.8 MB. Copyright © 2022 Piazza et al. 2022 Piazza et al. https://creativecommons.org/licenses/by/4.0/ This content is distributed under the terms of the Creative Commons Attribution 4.0 International license.

10.1128/mbio.02734-22.6 TABLE S2 KEGG pathway analysis of cluster 1 and cluster 2 proteins in Fig. S4. Download Table S2, PDF file, 0.5 MB. Copyright © 2022 Piazza et al. 2022 Piazza et al. https://creativecommons.org/licenses/by/4.0/ This content is distributed under the terms of the Creative Commons Attribution 4.0 International license.

10.1128/mbio.02734-22.7 TABLE S3 KEGG pathway analysis of differentially expressed ON proteins in MOB-513-p*dgcB* in the absence (−Mn) or presence (+Mn) of manganese. Download Table S3, PDF file, 0.7 MB. Copyright © 2022 Piazza et al. 2022 Piazza et al. https://creativecommons.org/licenses/by/4.0/ This content is distributed under the terms of the Creative Commons Attribution 4.0 International license.

### The Mn(II) oxidation phenotype in MOB-513 can be inhibited by enhancing the expression of the PilZ domain protein.

**FIG 8:**
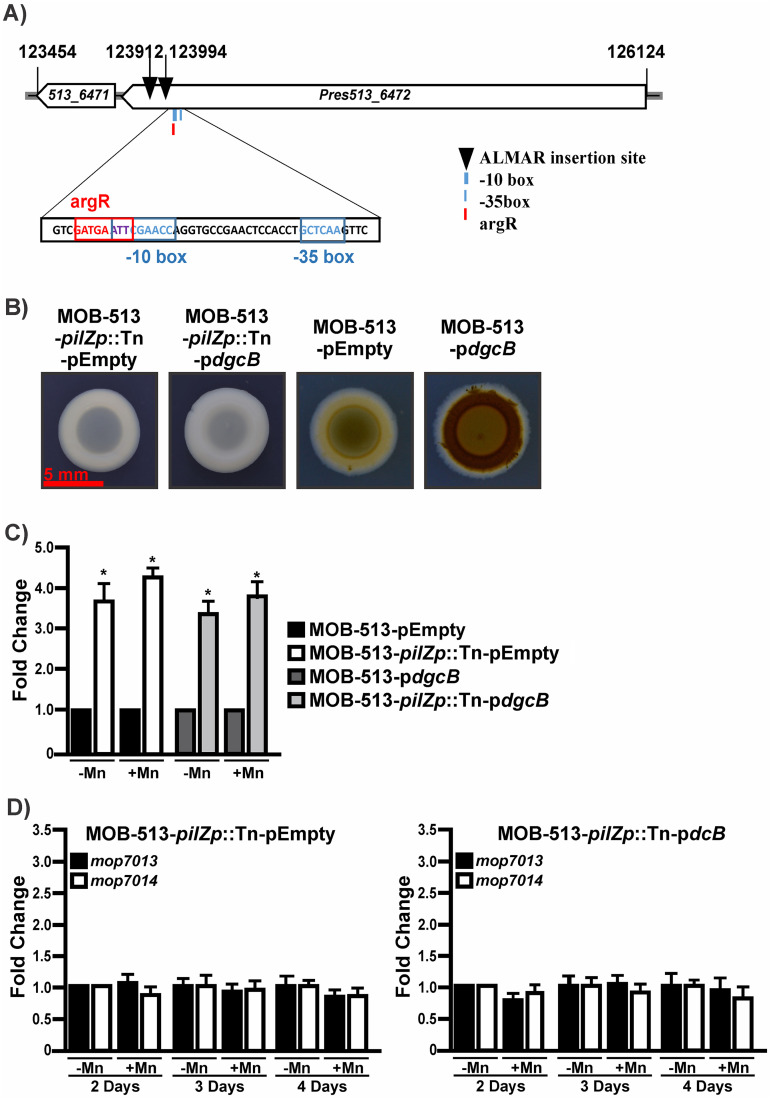
(A) Schematic representation of Tn insertion in the chromosomal region centered on the genes *Pres513_6471* and *Pres513_6472* from *P. resinovorans* strain MOB-513. The scheme is drawn to scale. Arrows pointing to the left indicate that genes were on the reverse strand. Dark arrowheads indicate ALMAR3 insertions at the corresponding nucleotide position in the chromosome. The sequence of the predicted promoter regions for *Pres_6471* in the arrangement described above is also shown. The −35 and −10 motifs inferred for the *Pres513_6471* promoter (blue boxed) and a putative *argR* motif (red boxed) were predicted using BPROM (SoftBerry). (B) Mn(II) oxidation in macrocolony biofilms of MOB-513-pilZp::Tn-pEmpty, MOB-513-*pilZp*::Tn-p*dgcB*, MOB-513-pEmpty, and MOB-513-p*dgcB*, used as a positive control for Mn(II) oxidation. Macrocolonies were set on Lept-Mn plates, incubated at 28°C, and imaged at 4 days. Red scale bar, 5 mm. (C) Expression of the *pilZ* gene in MOB-513-pEmpty, MOB-513-p*dgcB*, MOB-513-*pilZp*::Tn-pEmpty, and MOB-513-*pilZp*::Tn-p*dgcB*. Macrocolonies of the strains were grown on Lept and Lept-Mn and subjected to qRT-PCR assays to analyze the expression of *pilZ*. The *rpoD* gene was used as internal control for the calculation of relative gene expression. Bars indicate the expression levels of the *pilZ* gene in MOB-513-*pilZp*::Tn-pEmpty and MOB-513-*pilZp*::Tn-p*dgcB* relative to the expression levels in MOB-513-pEmpty and MOB-513-pdcB, respectively. (D) Expression of *mop7013* and *mop7014* genes in MOB-513-*pilZp*::Tn-pEmpty (left panel) and MOB-513-*pilZp*::Tn-p*dgcB* (right panel) strains. Macrocolonies of the strains were grown on Lept and Lept-Mn and subjected to qRT-PCR assays to analyze the expression of *mop7013* and *mop7014*. The *rpoD* gene was used as internal control for the calculation of relative gene expression. Bars indicate the expression levels of the genes in Lept-Mn relative to the expression levels in the absence of Mn(II). For panels C and D, values are the means of three biological replicates. Error bars indicate standard deviations. Data were analyzed by Student's *t* test, and asterisks indicate significant differences (*P* < 0.05) between samples and the control strain.

To further investigate the role of c-di-GMP in Mn(II) oxidation, we conducted a transposon mutagenesis in MOB-513 using the ALMAR3 transposon and screened the resulting library of mutants to search for those mutants that formed white, non-Mn(II)-oxidizing colonies in Lept-Mn agar plates. We detected the presence of two independent transposons inserted upstream of the start codon of the gene encoding the PilZ domain protein that were found to be differentially expressed in the proteomic assays (Fig. 8A). Sequence analysis revealed that ALMAR3 insertions were located 115 and 197 bp upstream of the gene encoding the PilZ domain protein and downstream of its predicted promoter region (Fig. 8A), and the resulting strains, named MOB-513-*pilZp*::Tn, were unable to oxide Mn(II).

Since the DgcB overexpression resulted in elevated c-di-GMP levels that accelerated and increased Mn(II) oxidation in MOB-513, we tested the effect of DgcB overexpression in the MOB-513-*pilZp*::Tn background. Also, MOB-513-*pilZp*::Tn was transformed with pEmpty as a control. Remarkably, the strain MOB-513-*pilZp*::Tn-p*dgcB* was also unable to oxidize Mn(II), which clearly contrasted with the strong Mn(II)-oxidizing phenotype exhibited by strain MOB-513-p*dgcB* (Fig. 8B).

Since insertions of the transposon either within or upstream of a gene may drive overexpression of the downstream gene by initiating expression from the transposon promoter, we analyzed the expression of the gene encoding the PilZ domain protein by quantitative real-time PCR (qRT-PCR). As shown in Fig. 8C, transposon insertion resulted in increased transcript levels of this gene, both in the presence and the absence of Mn(II), compared to the control strains (Fig. 8C). Note that while representative data for one of the mutants are shown, the results for both independent transposon mutants were the same in all the assays. Both transposon insertions disrupted the end of the *Pres513_6472* gene (178 bp), where *Pres513_6471* promoter was localized (Fig. 8A). This gene encodes a thymidine phosphorylase involved in pyrimidine salvage; the enzyme’s function is primarily catabolic (29). Therefore, the main effect of the transposon insertion on Mn(II) oxidation may be due to the overexpression of the PilZ domain protein.

Overall, these results suggested that the PilZ domain protein would act in MOB-513 by regulating Mn(II) oxidation in a negative manner. Consistent with this, the induction of expression of *mop7013* and *mop7014* genes observed in MOB-513-pEmpty and especially in MOB-513-p*dgcB* (Fig. 5A and B) was found not to occur at any time point tested in MOB-513-*pilZp*::Tn-pEmpty or MOB-513-*pilZp*::Tn-p*dgcB* (Fig. 8D). This confirmed that the PilZ domain protein is crucially involved in the process of Mn(II) oxidation conducted by MOB-513 and implied a role for c-di-GMP and Mop proteins in this process.

### Overexpression of DgcB in MOB-513 improved lyophilization and groundwater Mn(II) oxidation.

Previous studies showed the usefulness of MOB-lyophilized cultures to replace large volumes of inoculum and to enhance groundwater Mn removal performance (10). Since MOB-513-p*dgcB* shows a higher biofilm formation capacity and is a hyperoxidant bacterial strain, we expanded our analysis to consider the biotechnological relevance of c-di-GMP function in MOB-513. We determined that high c-di-GMP levels improved the lyophilization performance of MOB-513 (Table 3). In addition, for both MOB-513-pEmpty and MOB-513-p*dgcB*, the higher the initial content of BMnOx present in the cultures, the higher the observed survival rate (SR) (*P* < 0.05) (Table 3). Next, we analyzed the capacities of MOB-513-pEmpty, MOB-513-p*dgcB*, and MOB-513-p*pdeA* fresh cultures and lyophiles immobilized on sands to oxidize Mn(II) present in groundwater. Sand inoculated with MOB-513-p*dgcB* fresh cultures or lyophiles, both grown in Lept or Lept-Mn, showed a higher Mn(II) oxidation capacity than those inoculated with MOB-513-pEmpty (Table 3). On the other hand, and as previously observed (10), bacterial adaptation to Mn(II) performed by growing the strains in Lept-Mn enhanced the oxidation of Mn(II) present in groundwater (Table 3). No BMnOx was detected for MOB-513-p*pdeA* at the assayed time points (Table 3).

**TABLE 3:** Quantification of BMnOx production, bacterial growth, and lyophilization survival ratios of MOB-513-pEmpty, MOB-513-p*dgcB*, and MOB-513-p*pdeA* static cultures grown in Lept and Lept-Mn and results of analysis of groundwater Mn(II) oxidation by bacteria-inoculated sands

Quantification	MOB-513-pEmpty		MOB-513-p*dgcB*		MOB-513-p*pdeA*	
	Lept	Lept-Mn	Lept	Lept-Mn	Lept	Lept-Mn
Initial BMnOx in culture (μg/mL)	ND^*a*^	0.44 ± 0.08	ND	10.45 ± 0.30	ND	ND
Fresh culture (CFU/mL)	(2.50 ± 0.27) × 10^10^	(2.25 ± 0.24) × 10^10^	(6.50 ± 0.24) × 10^10^	(2.00 ± 0.22) × 10^11^	(1.65 ± 0.22) × 10^9^	(2.55 ± 0.22) × 10^9^
Lyophiles (CFU/mL)	(3.25 ± 0.34) × 10^7^	(3.60 ± 0.31) × 10^7^	(1.93 ± 0.20) × 10^8^	(2.33 ± 0.27) × 10^9^	(9.9 ± 0.25) × 10^5^	(1.79 ± 0.24) × 10^6^
SR (%)	0.130	0.160	0.296	1.165	0.06	0.07
Groundwater BMnOx (μg/mL) produced by:						
Fresh cultures adhered to sands	1.25 ± 0.10	6.72 ± 0.25	15.98 ± 0.33	23.92 ± 0.41	ND	ND
Lyophiles adhered to sands	1.28 ± 0.09	6.46 ± 0.31	17.90 ± 0.35	24.10 ± 0.42	ND	ND

a ND, not detected.

## DISCUSSION

The presence of Mn(II) in groundwater impacts human health negatively unless it is appropriately treated. Biological sand filter technology is widely used for groundwater potabilization as an eco-friendly strategy that does not require chemical addition, increases groundwater treatment capacity, and reduces operative costs (2). Biofilters harbor microbial communities recruited by the groundwater to be treated or by bioaugmentation approaches (4–8). Bioaugmentation using bacteria with high biofilm-forming and Mn(II)-oxidizing capabilities provides an inexpensive, simple, and efficient strategy for immobilizing these bacteria in the sand filters and optimizing Mn removal (4, 5, 8). Although many efforts have been made toward isolating bacteria with these characteristics (11), no studies have been performed to assess the importance of a biofilm lifestyle to the Mn(II) oxidation process.

The role of c-di-GMP in biofilm formation and in numerous bacterial functions, such as motility, regulation of cell cycle, differentiation, and virulence, is relatively well understood (13). In this work, we expanded these functions to describe a novel role for this second messenger in the regulation of Mn(II) oxidation in the environmental *P. resinovorans* strain MOB-513, isolated from sand biofilters that currently remove groundwater Mn with high efficiency (11). Our data shows that high levels of c-di-GMP increase both biofilm formation and Mn(II) oxidation capacities in MOB-513. Moreover, c-di-GMP-driven oxidation occurs by enzymatic activity and affects the abundance of Mop proteins previously linked to Mn(II) oxidation in other bacteria (19, 25, 30).

In macrocolony biofilms, oxygen becomes limited for cells located deeper into the biofilm section due to its consumption by those cells closer to the air interface, as shown by microsensor measurements of intact macrocolony biofilms, which revealed oxygen was depleted and became undetectable at a depth of ~70 μm in P. aeruginosa strain PA14 (31). The opposite occurs with the nutrients that are more abundant and available for biofilm cells located closer to the nutrient-providing agar. These differences create a clear stratification of cell metabolic activities across a biofilm (27, 28, 32). In this work, we showed for the first time evidence of stratified Mn(II) oxidation carried out by specific layers of cells in MOB-513 macrocolony biofilms, possibly due to the different metabolic conditions to which the cells were exposed. Remarkably, the levels of the second messenger c-di-GMP played a key role in defining the temporal and spatial localization of Mn(II) oxidation within the biofilm. In our study, cross-sections of MOB-513 macrocolonies were collected in the central region of the biofilms at a depth of ~100 μm which, based on the PA14 model (31), led us to assume that the bottom third of the MOB-513 macrocolony is anoxic. We found that Mn(II) ions were oxidized at different depths depending on the c-di-GMP levels: in macrocolonies of MOB-513 wild-type (control strain) and of the strain overexpressing PdeA, BMnOx accumulation occurred only in the upper, i.e., more-oxygenated subzone of the biofilms, whereas in the strain overexpressing DgcB, BMnOx additionally accumulated in a lower microoxic or anoxic subzone of the biofilms.

Our results showed that Mop enzymes are involved in Mn(II) oxidation in *P. resinovorans* MOB-513 biofilms and that the expression of these enzymes is positively regulated by Mn(II). Accordingly, an increase of the levels of the MopA protein in *A. manganoxydans* strain SI85-9A1 was observed in the presence of this metal (25). Interestingly, higher c-di-GMP intracellular levels accelerated, and enhanced *mop* gene expression and our proteomic data corroborated this. MCOs gene expression was not Mn(II) or c-di-GMP dependent; however, at this point, we cannot rule out the possibility that these or other Mn oxidases play a role in MOB-513 Mn(II) oxidation. The analysis of spatial expression of *mop* genes in MOB-513-p*dgcB* macrocolony biofilms showed that these genes express more intensely in those subzones where coincidentally Mn(II) oxidation takes place. c-di-GMP appears to be the key regulatory factor determining this spatial distribution of *mop* expression, as for MOB-513-pEmpty biofilms the *mop* expression pattern was more attenuated and rather homogenous, even in the presence of Mn(II). While basal and homogeneous expression levels of *mop* genes in MOB-513-pEmpty macrocolonies appeared to be sufficient to drive Mn(II) oxidation in the upper biofilm layer, the enhanced BMnOx accumulation in this layer and in an additional bottom layer in MOB-513-*pdgcB* macrocolonies most likely required additional c-di-GMP-mediated induction of Mop proteins. Future studies will be needed to clarify how c-di-GMP promotes this striking spatial pattern of Mn(II) oxidation.

High c-di-GMP levels and the presence of Mn(II) extensively remodeled the MOB-513 proteome. Among the upregulated proteins for MOB-513 in the presence of Mn(II), we observed a GGDEF/EAL domain protein, suggesting a potential role in changing the intracellular c-di-GMP levels in response to this metal. Furthermore, we identified Pres513_6471, a PilZ domain protein that was upregulated for MOB-513-p*dgcB* in the presence of Mn(II). The PilZ domain is a c-di-GMP-binding protein domain with a remarkably wide range of binding affinities to regulate different pathways (33). Single-domain PilZ proteins are widespread, and several of them mediate cellular functions and bacterial behaviors known to be regulated by c-di-GMP (34). The upregulation of this protein in MOB-513-p*dgcB* in the presence of Mn(II) suggests a role in Mn(II) oxidation signaling by c-di-GMP. Our screening for transposon mutants that lose their ability to oxidize Mn(II) led us to identify two mutants with the transposon inserted upstream of the start codon of the gene encoding the PilZ domain protein whose expression was found altered in the proteomic assays. We expected that at least some mutations that rendered the cells unable to oxidize Mn(II) occurred in signaling proteins found to be upregulated by c-di-GMP in the proteomic assay. Less predictable, but consistent with the insertion of the transposon upstream of a gene, the two mutants here identified showed overexpression of the gene encoding the PilZ domain protein. Such overexpression correlated not only with complete inhibition of MOB-513 Mn(II) oxidation, but also with the absence of induction of *mop7013* and *mop7014* expression. Since this PilZ domain protein was found in the proteomic analysis to be upregulated in the MOB-513 derivative that overexpressed DgcB, it may be the case that such upregulation serves as a compensatory mechanism that cells deploy to cope with the unnatural situation of having highly elevated c-di-GMP levels in their cytosol. Therefore, these results indicate that the PilZ domain protein is involved in both c-di-GMP signaling and Mn(II) oxidation and that its actual mechanism of action deserves further investigations.

Bacteria can use Mn(II) oxidation as an adaptive advantage to survive adverse environmental conditions (16), and several advantages of possessing Mn(II)-oxidizing activity for biofilms may be considered. Previous reports indicated that Mn(II) oxidation increases tolerance to oxidative stress (35), and extracellular insoluble accumulation of BMnOx can prevent predation or viral attack (16), can protect from radiation (36), and can enable the oxidative degradation of natural organic matter, with bacteria gaining energy from this process (37). Our results showed that the second messenger c-di-GMP triggered both biofilm formation and Mn(II) oxidation in MOB-513, suggesting that Mn(II) oxidation evolved as an adaptation to aid survival within the biofilm. Mn(II) oxidation and biofilm formation may act simultaneously to some extent, with c-di-GMP determining the spatial and temporal distribution of BMnOx across the MOB-513 biofilm.

Finally, we expanded the analysis to consider the biotechnological relevance of c-di-GMP Mn(II) oxidation control in MOB-513. We examined its impact on lyophilization and the Mn(II) oxidation efficiency of lyophiles, with our data showing that high levels of c-di-GMP correlated with higher lyophilization efficiencies and higher groundwater Mn(II) oxidation capacities of MOB-513 lyophiles. Overall, these results provide evidence to support the role of c-di-GMP in biofilm formation and Mn(II) oxidation in *P. resinovorans* and provide new strategies for optimizing the biotechnological application of this bacterium in bioremediation.

## MATERIALS AND METHODS

### Plasmids, strains, and growth conditions.

The plasmids and bacterial strains used in this study are listed in Table 2. Proteins DgcB (gene BB3903) and PdeA (gene BB2664) from Bordetella bronchiseptica (21, 22) were overexpressed by using the vector pBBR1MCS-5 under the control of the constitutive promoter *nptII* (38). These plasmids and the corresponding empty vector (pEmpty) were transformed into *P. resinovorans* MOB-513 by electroporation (39). The plasmid p*WspR19* corresponds to the pBBR1MCS-5 vector containing a constitutively active allele of the DGC WspR (23).

For the construction of promoter-*gfp* reporter fusions, the predicted promoters of genes *mop7013* and *mop7014* were cloned (primers are described in Table 4) upstream of the promoterless *gfp* gene in the pPROBE-KT vector (40).

**TABLE 4:** Oligonucleotides used in this study

Purpose	Oligonucleotide	sequence (5′–3′)
qRT-PCR	7013-F	TTCGGGCAGTTCTTCGACC
	7013-R	CGTCAGGACCATGGGTGAT
	7014-F	GAAGGAAATGGCTCCCTTCA
	7014-R	GAATACCGCCGTGTCATAGTT
	5806-F	TTGGCACGGATCAGAATGC
	5806-R	GGTGGTGATGCCATTGAAG
	mcoA-F	GGCCAGGGCTACAACATG
	mcoA-R	CACCGTTCTTGATCTTCC
	mnxG-F	ACTACATCGACGCCCAGGG
	mnxG-R	GAAGTGGCAGTGGTAGATG
	ropD-F	GACGACGAAGAAGACGAAGAAG
	rpoD-R	ATCGGAAACAGCGGTGAAG
	pilZ-F	CTTCATGCTGCTGGGAGAAA
	pilZ-R	GCAGTTGCGTTCGTCCT
Promoter cloning in pPROBE-KT	7013p-F	AATGCAAGCTTGAACGGTCAGGACAGTATTACC
	7013p-R	CTGAGGATCCGAAGTTGGCCATCTTTATGTC
	7014p-F	AATGCAAGCTTATTATGAGAGGGCCGCTTC
	7014p-R	CTGAGGATCCGAAGTTGGCCATATTTGTTTCC
Locating transposition insertion site	Arb-PCR	CGCAAACCAACCCTTGGCAG
	Arb1	GGCCAGCGAGCTAACGAGAC
	Arb1b	GGCCAGCGAGCTAACGAGACNNNNGATAT
	Almar3-seq	ACATATCCATCGCGTCCGCC

*P. resinovorans* MOB-513 strains were grown at 28°C in either Luria-Bertani (LB) or in Lept medium with or without supplementation of 100 μM MnCl_2_ (Lept-Mn or Lept) (41). Escherichia coli and P. aeruginosa strains were grown in LB medium at 37°C. When required, the following antibiotics at the specified concentrations were used: kanamycin (Km), 20 μg/mL; chloramphenicol (Cm), 10 μg/mL; gentamicin (Gm), 10 μg/mL.

### Bacterial genome sequencing and bioinformatics techniques.

*P. resinovorans* MOB-513 genomic DNA was sequenced using the Illumina technology platform (Illumina Inc. USA) as previously described (42).

### Quantification of intracellular c-di-GMP concentrations.

The quantification of c-di-GMP levels was performed using the cyclic-di-GMP assay kit from Lucerna (catalog number 200-100). Cultures of the strains were adjusted to optical density at 600 nm (OD_600_) of 0.2 and set up for the assay in 30-μL aliquots along with assay reagents and serially diluted c-di-GMP standards, and then the c-di-GMP concentration was calculated according to the standard calibration curve. Appropriate sample dilution factors were multiplied to get the final c-di-GMP concentrations (in picograms per microliter).

### Biofilm formation assays.

Biofilm formation by MOB-513 strains was assessed as described previously (11). Briefly, bacterial cultures were grown in LB medium with shaking (200 rpm) until exponential growth phase, standardized at the same OD_600_ of 1.5, and then diluted 1:10 in fresh LB medium. A total of 2 mL of these bacterial suspensions was placed in borosilicate glass tubes and incubated statically at 28°C. After 7 days of incubation, planktonic cells were removed by washing, and biofilms formed at the air-liquid interface were quantified by crystal violet (CV) staining. Macrocolony biofilms of MOB-513 strains were set up as previously described (24). Briefly, 5 μL of an overnight culture grown in LB and adjusted to an OD_600_ of 0.1 was spotted onto Lept or Lept-Mn agar plates and incubated at 28°C for up to 7 days.

### Motility assays.

For each MOB-513 strain, 5 μL of an overnight culture adjusted to an OD_600_ of 1.5 was spotted onto the center of an LB swimming plates (0.3% agar) as previously described (43), and migration zones after 72 h of incubation were measured.

### BMnOx quantification.

Mn(II) oxidation was quantified by using leucoberbelin blue (LBB) dye solution as previously described (11).

### Cryosectioning of macrocolony biofilms and bright-field and fluorescence microscopy.

The procedures for cryoembedding and cryosectioning macrocolony biofilms of MOB-513 strains into 5-μm-thin cross-sections were carried out as previously described (24). Five-micrometer-thick macrocolony sections perpendicular to the plane of the macrocolony were sectioned using a cryostat (Thermo Fisher Scientific) set at 20°C and using disposable Sec35 blades (Thermo Fisher Scientific). The sections were placed on microscope slides and mounted with Mowiol 4-88. To examine the expression patterns in cross-sections, at least 3 individual macrocolony biofilms per condition were analyzed, and a minimum of 10 cross-sectional images of each single macrocolony were observed and captured.

### Quantification of Mn(II) oxidase activity.

Mn(II) oxidase activity of crude total protein extracts (PE) of MOB-513 strains was evaluated by quantifying the conversion reaction of Mn(II) to BMnOx *in vitro* using LBB (11). Macrocolonies were grown on either Lept (as control) or Lept-Mn agar plates. At different time points (days), cells from individual macrocolony biofilms were collected, washed once with buffer (10 mM HEPES [pH 7.5]), resuspended in 1 mL of the same buffer supplemented with 1 mM phenylmethylsulfonyl fluoride, and then subjected to sonication on ice with a 3-mm probe with six cycles of 10 s on and 1 min off with a 25% amplitude (Sonics Materials VC 750 ultrasonic processor). Samples were centrifuged at maximum speed at 4°C, and supernatants were transferred to a new tube. Total proteins in these extracts (PE) were quantified by the Bradford method. For assaying Mn(II) oxidase activities, 1 mL of reaction mixture (10 mM HEPES [pH 7.5], 0.020 mg/mL PE, 5 mM MnCl_2_) was incubated statically at 28°C for about 24 h. The effect of Ca(II) or Cu(II) was studied by adding 25 mM CaCl_2_ (25) or 0.4 mM CuSO_4_ (26) to the reaction mixtures. Control reaction mixtures were heated at 95°C for 15 min. Values of Mn(II) oxidase activity were normalized by the activity measured for each reaction mixture at time zero (*t*_0_), i.e., before the incubation at 28°C.

### RNA preparation and quantitative real-time PCR.

RNA was extracted from pools of macrocolonies using 1 mL TRIzol reagent (Invitrogen) according to the manufacturer’s instructions and subjected to DNase (Promega) treatment, and cDNA was synthesized using Moloney murine leukemia virus reverse transcriptase (Promega, USA). Gene-specific primers were used (Table 4). Then, qRT-PCRs were performed in a Mastercycler Realplex thermal cycler (Eppendorf) using Platinum *Taq* DNA polymerase (Invitrogen) and SYBR green I (Roche) to monitor double-stranded DNA synthesis. The *rpoD* gene was used as internal control (44).

### Quantification of GFP fluorescence in macrocolony cells.

Quantification of GFP fluorescence in macrocolonies of MOB-513 coexpressing DgcB was assayed as previously described (45). Briefly, 2-day-old macrocolonies of MOB-513 strains grown on Lept or Lept-Mn were collected, washed, and resuspended in phosphate-buffered saline (PBS) to achieve an OD_600_ of 0.5. Aliquots of 200 μL of each cell suspension were placed into 96-well flat-bottom black plates (Greiner Bio-One). Fluorescence intensity (*F*) and the final OD_600_ of each sample was recorded on a Synergy 2 multimode microplate reader (BioTek) using excitation and emission filters with wavelengths of 485 ± 20 nm and 535 ± 20 nm (± standard deviation [SD]), respectively. *F* values were normalized by applying the following formula: *F* = [RFU(1)/OD_600_(1)] − [RFU(2)/OD_600_(2)], where RFU(1) and OD_600_(1) are the fluorescence intensity measured by instrument and the final OD_600_ determined for the strain expressing a promoter-*gfp* fusion, respectively, and RFU(2) and OD_600_(2) are the same parameters determined for the sample of cells carrying the empty vector (pPROBE-KT).

### Proteomic assays and label-free quantification.

Twelve macrocolony biofilms of MOB-513 strains were grown for 2 days on Lept or Lept-Mn agar plates. Then, they were scraped, resuspended in PBS, and subjected to protein extraction. Samples containing 30 μg of whole-cell proteins were sent to the Proteomics Core Facility of CEQUIBIEM, where protein digestion and mass spectrometry analysis were performed by with a nano-high-performance liquid chromatography system coupled to a mass spectrometer with Orbitrap technology.

### Random transposon mutagenesis.

MOB-513 was transformed using the mariner plasmid pALMAR3 as described previously (46). Transformants were initially selected on LB supplemented with tetracycline. Transposon insertions resulting in the loss of Mn(II) oxidation capacity behavior were screened on Lept-Mn plates. The location of the transposon insertion in each case was determined by arbitrary PCR (47) using the primer pair Arb1b and Arb-PCR for the first PCR round and the primer pair Arb1 and Almar3-seq for the second PCR round. The final PCR products were sequenced using the Almar3-seq primer.

### Bacterial lyophilization and quantification of bacterial survival ratios and BMnOx production.

MOB-513 strains were lyophilized as previously described (10). Viability of bacteria (in CFU per milliliter) before and after lyophilization was used to calculate the cell SR (a percentage), as follows: SR = (CFU after lyophilization/CFU before lyophilization) × 100.

### Quantification of oxidation of Mn(II) present in groundwater.

The oxidation of Mn(II) present in groundwater carried out by sand-immobilized bacteria derived from fresh cultures and lyophiles was quantified as previously described (10).

### Statistical analysis.

Quantifications were performed from three biological replicates (in technical triplicates). Means and SD are shown in the figures. All data were statistically analyzed using one-way analysis of variance (ANOVA; *P* < 0.05), by Tukey’s test, or by Student's *t* test (as indicated in the figure legends).

### Data availability.

The assembled genome of *P. resinovorans* MOB-513 was deposited at DDBJ/ENA/GenBank under accession number JAJOHH000000000.
