# Supplementary figures and images for: Cyclic di-GMP Signaling Links Biofilm Formation and Mn(II) Oxidation in Pseudomonas resinovorans
Source: mBio. 2022 Nov 14;13(6):e02734-22. doi: 10.1128/mbio.02734-22 (PMC9765421; doi:10.1128/mbio.02734-22)

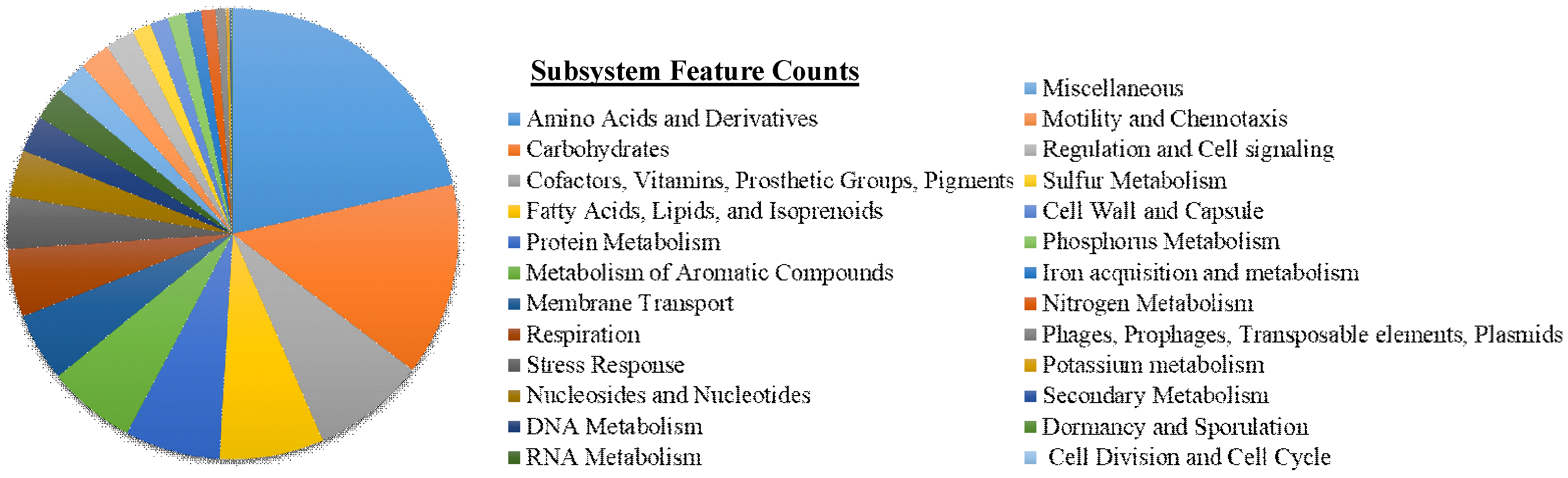

Supplement: FIG S1 [file mbio.02734-22-s0001.tif]

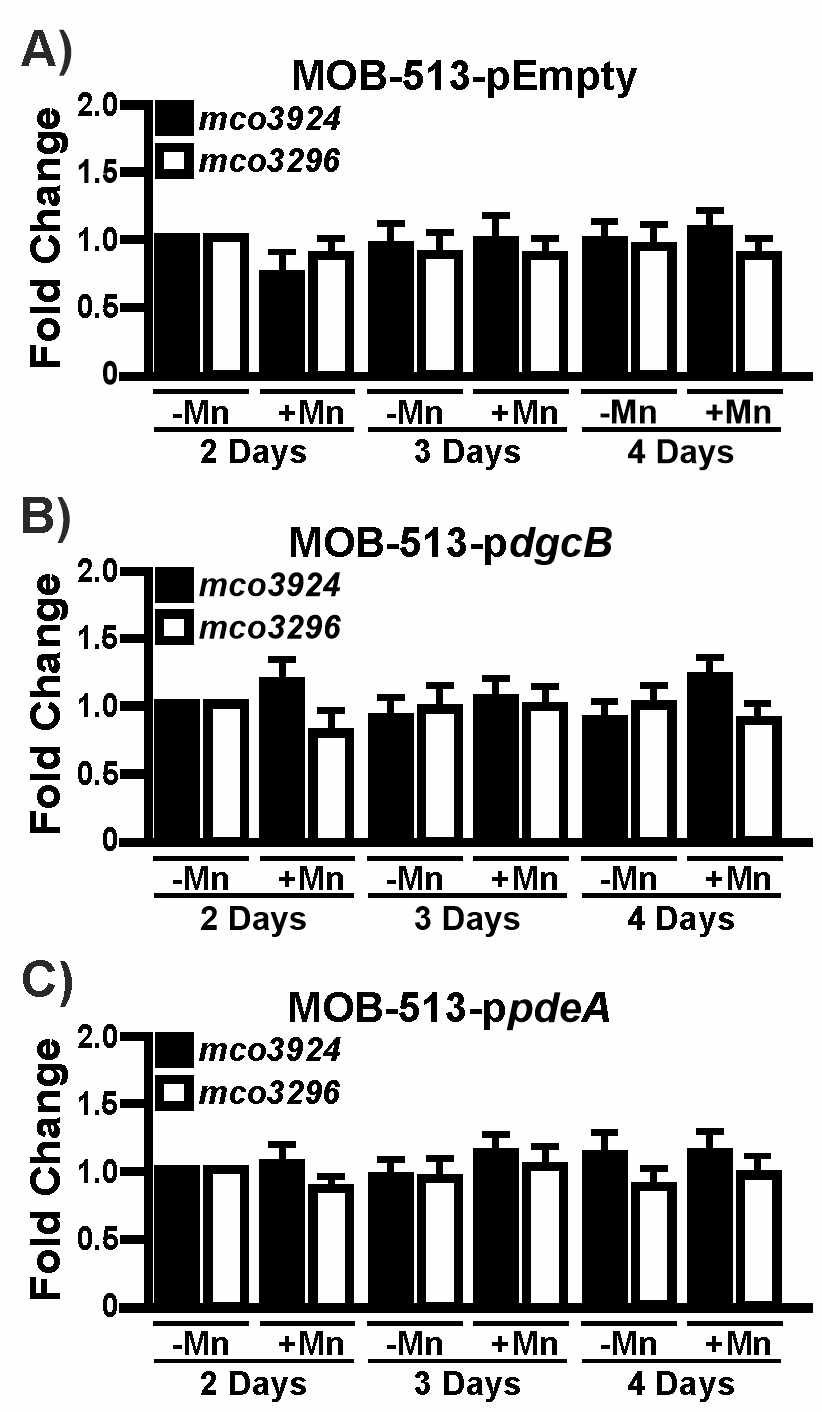

Supplement: FIG S2 [file mbio.02734-22-s0002.tif]

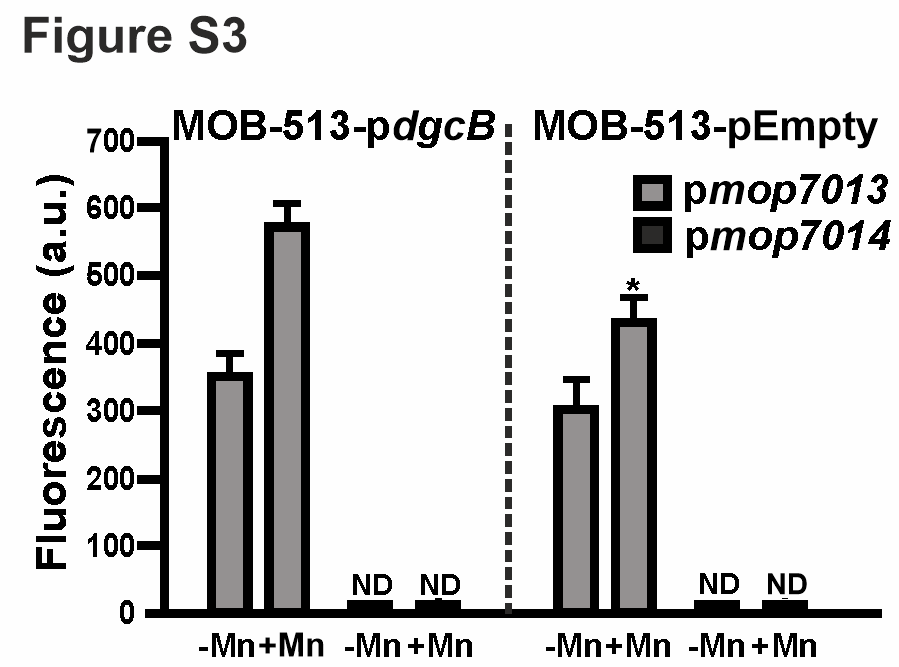

Supplement: FIG S3 [file mbio.02734-22-s0003.tif]

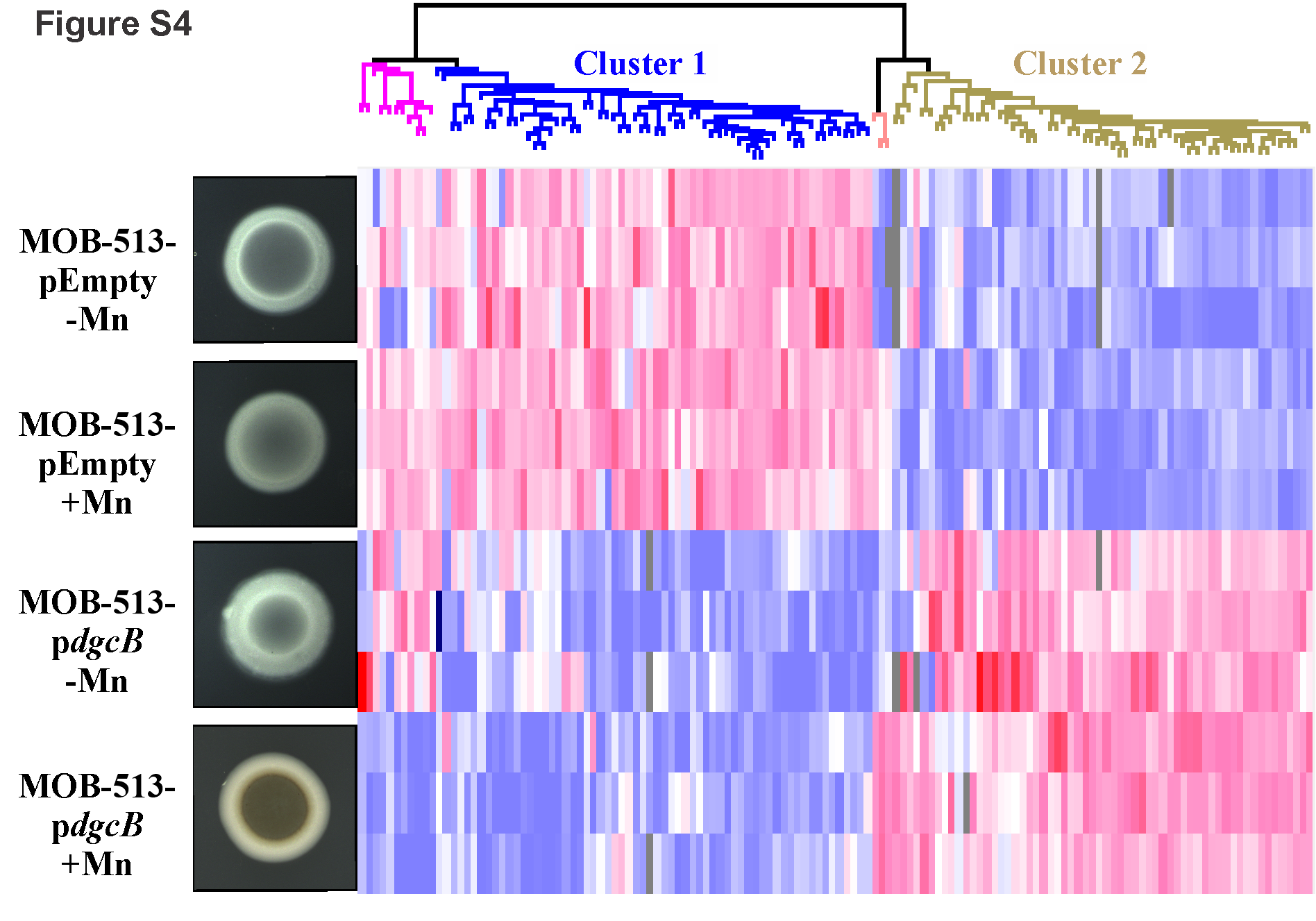

Supplement: FIG S4 [file mbio.02734-22-s0004.tif]
