# Supplementary material for: Cyclic di-GMP Signaling Links Biofilm Formation and Mn(II) Oxidation in Pseudomonas resinovorans
Source: mBio. 2022 Nov 14;13(6):e02734-22. doi: 10.1128/mbio.02734-22 (PMC9765421; doi:10.1128/mbio.02734-22)
Supplement: TEXT S1 [file mbio.02734-22-s0005.pdf]

Proteomic Supplementary Information.

Label free quantification was conducted on proteomes from 2-day-old macrocolonies of MOB-513-pEmpty and MOB-513-p*dgcB*.

First, in order to corroborate the overexpression of *dgcB*, the peptides data obtained were searched against the UniProt *B. bronchiseptica* protein database. Only one protein (GGDEF domain-containing protein, OS=*B. bronchiseptica*) was identified and it was present in the strain MOB-513- p*dgcB* grown both in the absence and presence of Mn(II) (data not shown), consistent with the overexpression of *dgcB*.

Proteomic data showed that 1,912 proteins were detected in both treatments, Lept in absence (-Mn) or presence (+Mn) of Mn, in at least two out of three replicates each. Hierarchical cluster analysis of proteomic data was performed to group the proteins according to their differential abundance. The heatmap evidences the presence of two main clusters in which protein abundance was either anticorrelated ("cluster 1") or correlated ("cluster 2") with the production of c-di-GMP, independently of the presence or absence of Mn in the medium (Fig. S6; see also Table S2).

A total of 62 proteins contained in the **Cluster 1** were downregulated by c-di-GMP and 42 proteins were assigned to Kegg-Biological Process categories (Table S2). It should be noted that the most strongly down-regulated proteins in the MOB-513-p*dgcB* proteome were associated with the Genetic Information Processing, Amino acid metabolism, Microbial metabolism in diverse environments and Biosynthesis of secondary metabolites processes. Furthermore, as it was expected, bacterial motility proteins such as the Twitching motility protein PilT (Pres513_322); the Type IV fimbria assembly, ATPase PilB, named (Pres513_6341); and the Chemotaxis response- phosphatase CheZ (Pres513_6884), were affected negatively by the over-expression of *dgcB*.

On the other hand, 60 proteins were contained in the **Cluster 2** and 35 were assigned to specific Kegg-Biological Processes (Table S2). There was a significant positive correlation between over-expression of *dgcB* and proteins related to Genetic information processes and the most intriguing correlation is with the overexpression of ribosomal proteins. Furthermore, there was an increment in proteins like chaperons, phosphorelay signal transduction system and cold acclimation. Over-expression of *dgcB* showed the upregulation of the CpsC protein (Pres513_2487) and YccZ (Pres513_2459) involved in polysaccharide export, since polysaccharide contribute to the structural stability and protection of biofilms as well as to the retention of water and nutrients (Flemming and Wingender, 2010), this result is in agreement with the observations that MOB-513-p*dgcB* produced more biofilm than MOB-513-pEmpty.

Besides the proteins analysed by the hierarchical cluster heat map, a set of proteins that were ON and OFF were also found in the analysis of differentially expressed proteins by over-expression of *dgcB* (Table S3). Among the proteins differentially expressed in the absence of Mn(II), 11 were detected only in MOB-513-pEmpty and 9 were exclusive of MOB-513-*p dgcB*. Out of these 20 proteins, 14 were assigned to Kegg-Biological Process categories. In the presence of Mn, 23 proteins were present only in MOB-513-pEmpty and 5 proteins were ON in MOB-513-p*dgcB*. Out of them, a total of 16 proteins were associated with Kegg-Biological Process categories (Table S3).

On the other hand, differentially expressed proteins in response to Mn(II) in each strain were analysed. The proteome patterns of MOB-513-pEmpty in the presence or absence of Mn showed that a total of 13 proteins were Mn-upregulated >2-fold ($p<0.05$) and 20 proteins were “ON” while 6 proteins were Mn-downregulated <0.5-fold ($p<0.05$) and 5 proteins were “OFF” in the Mn treatment. Among these differentially expressed proteins, 22 could be

assigned to Kegg-Biological Process categories (Table S1). Regarding MOB-513-p*dgcB*, a total of 19 proteins were Mn-upregulated >2-fold ($p<0.05$) and 22 proteins were "ON," while 20 proteins were Mn-downregulated <0.5-fold ($p<0.05$) and 19 proteins were "OFF" in the Mn treatment. Among these differentially expressed proteins, 46 could be assigned to Kegg-Biological Process categories (Table S1). The proteomic profiles of both strains did not exhibit the overexpression of specific Kegg-Biological Process, showing the importance of Mn in enhancing enzymatic activity rather than in biological process regulation.
