# Supplementary material for: Cyclic di-GMP Signaling Links Biofilm Formation and Mn(II) Oxidation in Pseudomonas resinovorans
Source: mBio. 2022 Nov 14;13(6):e02734-22. doi: 10.1128/mbio.02734-22 (PMC9765421; doi:10.1128/mbio.02734-22)
Supplement: TABLE S1 [file mbio.02734-22-s0006.pdf]

Table S1. KEGG pathway analysis of Down- and Up-regulated proteins in MOB-513-pEmpty and MOB-513-p*dgcB* in the presence of Mn(II).

Locus Tag	Protein	Fold Change	Kegg Pathway name
	**Down-regulated in MOB-513-pEmpty in presence of Mn(II)**		
**Pres513_141**	Catalase KatE (EC 1.11.1.6)	OFF	BSM, CM, AM, ST, P
Pres513_184	Efflux ABC transporter, ATP-binding protein	<0.05	T
Pres513_523	hypothetical protein	<0.05	NA
**Pres513_2619**	Transcriptional regulator, AraC family	OFF	NA
Pres513_2878	Cys-tRNA(Pro) deacylase YbaK	<0.05	GIP
**Pres513_3417**	Protein related to penicillin acylase	OFF	BSM,
Pres513_3475	LSU ribosomal protein L29p (L35e)	<0.05	Ribosome, GIP
Pres513_4739	Cob(I)alamin adenosyltransferase (EC 2.5.1.17)	<0.05	MCV
**Pres513_4774**	hypothetical protein	OFF	NA
**Pres513_5377**	DOPA 4,5-dioxygenase (EC 1.14.99.-)	OFF	NA
Pres513_5535	Integration host factor beta subunit	<0.05	GIP
	**UP-regulated in MOB-513-pEmpty in presence of Mn(II)**		
**Pres513_417**	Stresses-induced protein Ves (HutD)	ON	NA
Pres513_544	Putative phosphatase	>2	NA
Pres513_731	Ferrichrome-iron receptor	>2	T
Pres513_1116	4-aminobutyraldehyde dehydrogenase (EC 1.2.1.19)	>2	MMDE, AM
Pres513_1344	Rho-specific inhibitor of transcription termination (YaeO)	>2	GIP
Pres513_1346	Nucleoside triphosphate pyrophosphohydrolase MazG (EC 3.6.1.8)	>2	NM, MCV
**Pres513_1676**	Multidrug efflux system MdtABC-TolC, membrane fusion component MdtA	ON	ST, T
**Pres513_1800**	Cyanoalanine nitrilase (EC 3.5.5.4)	ON	MMDE, EM, AM, XDM
**Pres513_2118**	3-oxoacyl-[acyl-carrier protein] reductase (EC 1.1.1.100)	ON	NM, AM
**Pres513_2269**	hypothetical protein	ON	NA
Pres513_2272	hypothetical protein	>2	NA
**Pres513_2273**	hypothetical protein	ON	NA
Pres513_2330	Lysophospholipase (EC 3.1.1.5); Monoglyceride lipase (EC 3.1.1.23)	>2	NA
**Pres513_2698**	TonB-dependent ferric achromobactin receptor protein	ON	T
**Pres513_3172**	Hybrid sensory histidine kinase in two-component regulatory system with EvgA	ON	ST
Pres513_3174	DNA-binding response regulator, LuxR family	>2	ST, AR
Pres513_3259	Ferrichrome-iron receptor	>2	T
**Pres513_3402**	Putative esterase EstA	ON	NA
**Pres513_3549**	hypothetical protein	ON	NA
Pres513_3629	Glycoprotein gp2	>2	NA
Pres513_3630	Glycoprotein gp2	>2	NA
**Pres513_4417**	Universal stress protein UspA and related nucleotide-binding proteins	ON	NA
**Pres513_4422**	UPF0061 protein YdiU	ON	NA
Pres513_4441	hypothetical protein	>2	NA
**Pres513_4541**	GGDEF domain/EAL domain protein	ON	NA
Pres513_4581	Alginate regulatory protein AlgP, positive transcriptional regulator of AlgD	>2	NA
**Pres513_4659**	serine/threonine protein kinase	ON	NA
**Pres513_5060**	hypothetical protein	ON	T
**Pres513_5762**	Putative activity regulator of membrane protease YbbK	ON	NA
**Pres513_6309**	Uncharacterized protein conserved in bacteria, NMA0228-like	ON	NA
**Pres513_6827**	Iron-sulfur cluster assembly iron binding protein IscA	ON	GIP
**Pres513_6854**	Exonuclease SbcD	ON	GIP
**Pres513_6972**	Cytochrome c heme lyase subunit CcmF	ON	T

Locus Tag	Protein	Fold Change	Kegg Pathway name
	**Down-regulated in MON-513-p*dgcB* in presence of Mn(II)**		
**Pres513_11**	DNA-binding response regulator KdpE	OFF	ST, QS
**Pres513_71**	CBS domain protein	OFF	NA
Pres513_89	Ribosomal large subunit pseudouridine synthase E (EC 5.4.99.20)	<0.05	GIP
Pres513_184	Efflux ABC transporter, ATP-binding protein	<0.05	T
**Pres513_775**	Lipopolysaccharide export system protein LptC	OFF	T
Pres513_813	16S rRNA (cytidine(1402)-2'-O)-methyltransferase (EC 2.1.1.198)	<0.05	GIP
Pres513_1010	Transcriptional regulator, AsnC family	<0.05	GIP
**Pres513_1102**	CheW domain protein WspB	OFF	ST, B
**Pres513_1186**	hypothetical protein	OFF	NA
Pres513_1377	hypothetical protein	<0.05	NA
**Pres513_1676**	Multidrug efflux system MdtABC-TolC, membrane fusion component MdtA	OFF	ST, T
**Pres513_1719**	Glutathione S-transferase (EC 2.5.1.18)	OFF	NA
Pres513_1731	Isoquinoline 1-oxidoreductase alpha subunit (EC 1.3.99.16)	<0.05	NA
Pres513_2110	Transcriptional regulator, GntR family	<0.05	GIP
**Pres513_2118**	3-oxoacyl-[acyl-carrier protein] reductase (EC 1.1.1.100)	OFF	NM, AM
**Pres513_2130**	D-amino acid dehydrogenase (EC 1.4.99.6)	OFF	AM
Pres513_2843	Type I secretion system ATPase @ Type I secretion system ATPase, LssB family LapB	OFF	T, SS
Pres513_2943	Acetate kinase (EC 2.7.2.1)	<0.05	MMDE, CM, EM, AM
**Pres513_2969**	Methyl-accepting chemotaxis sensor/transducer protein	OFF	ST, BMP
**Pres513_3215**	Ribosomal protein S6--L-glutamate ligase	OFF	NA
Pres513_3357	FIG002188: hypothetical protein	<0.05	NA
**Pres513_3417**	Protein related to penicillin acylase	OFF	BSM
Pres513_3489	DNA-directed RNA polymerase beta' subunit (EC 2.7.7.6)	<0.05	GIP
Pres513_3490	DNA-directed RNA polymerase beta subunit (EC 2.7.7.6)	<0.05	GIP
Pres513_3579	transcriptional regulator, Crp/Fnr family	<0.05	GIP
**Pres513_3903**	Cation transport ATPase	OFF	NA
Pres513_4411	Glutamate synthase [NADPH] large chain (EC 1.4.1.13)	<0.05	BSM, MMDE, BAM, EM, AM
**Pres513_4658**	Protein phosphatase 2C-like	OFF	NA
Pres513_4933	Apolipoprotein N-acyltransferase / Copper homeostasis protein CutE	<0.05	NA
**Pres513_5040**	Transcriptional regulator, GntR family	OFF	NA
Pres513_5215	hypothetical protein	<0.05	NA
**Pres513_5314**	Nitrogen regulation protein NR(I)	OFF	ST
Pres513_5863	SAM-dependent methyltransferase YafE (UbiE paralog)	<0.05	NA
Pres513_5955	RNA polymerase sigma factor RpoS	<0.05	B, GIP
Pres513_6213	Ribonuclease III (EC 3.1.26.3)	<0.05	GIP
Pres513_6302	Transcriptional regulator, GntR family domain / Aspartate aminotransferase (EC 2.6.1.1)	<0.05	GIP
**Pres513_6364**	hypothetical protein	OFF	NA
Pres513_6833	Inositol-1-monophosphatase (EC 3.1.3.25)	<0.05	BSM, CM, ST
**Pres513_6854**	Exonuclease SbcD	OFF	GIP
	**UP-regulated in MOB-513-p*dgcB* in presence of Mn(II)**		
**Pres513_417**	Stresses-induced protein Ves (HutD)	ON	NA
Pres513_424	Multidrug efflux system, membrane fusion component => MexV of MexVW-OprM	>2	AR, T, GIP
Pres513_544	Putative phosphatase	>2	NA
Pres513_731	Ferrichrome-iron receptor	>2	T
**Pres513_1099**	Signal transduction histidine kinase CheA	ON	B, ST
**Pres513_1617**	Pyruvate dehydrogenase E1 component (EC 1.2.4.1)	ON	BSM, MMDE, CM
**Pres513_1703**	DNA-binding response regulator ColR	ON	NA
Pres513_1726	Multidrug efflux system, inner membrane proton/drug antiporter (RND type) => MexF of MexEF-OprN system	ON	AR, T
**Pres513_1730**	Transcriptional regulator, AraC family	ON	NA
**Pres513_2224**	Malonate decarboxylase beta subunit	ON	NA
**Pres513_2269**	hypothetical protein	ON	NA
Pres513_2273	hypothetical protein	>2	NA
**Pres513_2552**	Transcriptional regulator, AraC family	ON	GIP
Pres513_2610	(2E,6E)-farnesyl diphosphate synthase (EC 2.5.1.10)	>2	BSM, MTP
Pres513_2698	TonB-dependent ferric achromobactin receptor protein	>2	T
**Pres513_3155**	Electron transfer flavoprotein, alpha subunit	ON	E
Pres513_3240	Long chain acyl-CoA dehydrogenase [fadN-fadA-fadE operon] (EC 1.3.8.8)	>2	NA
Pres513_3277	Spermidine/putrescine import ABC transporter substrate-binding protein PotD (TC 3.A.1.11.1)	ON	T
**Pres513_3402**	Putative esterase EstA	ON	NA
**Pres513_3505**	Glycolate dehydrogenase (EC 1.1.99.14), FAD-binding subunit GlcE	ON	BSM, MMDE, CM
Pres513_3629	Glycoprotein gp2	>2	NA
Pres513_3630	Glycoprotein gp2	>2	NA
**Pres513_3637**	Exodeoxyribonuclease V beta chain (EC 3.1.11.5)	ON	GIP
Pres513_4328	Tryptophan synthase (indole-salvaging) (EC 4.2.1.122)	>2	NA
Pres513_4503	Thioredoxin	>2	NA
**Pres513_4687**	hypothetical protein	ON	NA
**Pres513_5226**	Low molecular weight protein tyrosine phosphatase (EC 3.1.3.48)	ON	ST
Pres513_5293	Glucose-6-phosphate 1-dehydrogenase (EC 1.1.1.49)	>2	BSM, MMDE, AM, CM, E
Pres513_5339	tRNA-5-carboxymethylaminomethyl-2-thiouridine(34) synthesis protein MnmE	>2	GIP
Pres513_5553	Tripartite tricarboxylate transporter TctC family	>2	ST, T
**Pres513_5632**	Similar to citrate lyase beta chain, 3	ON	CM
**Pres513_5762**	Putative activity regulator of membrane protease YbbK	ON	NA
**Pres513_6005**	Gliding motility-associated ABC transporter ATP-binding protein GldA	ON	T
Pres513_6187	Uncharacterized protein YqcC	>2	NA
**Pres513_6334**	FAD/FMN-containing dehydrogenases	ON	NA
Pres513_6471	hypothetical protein (PilZ domain-containing protein)	>2	NA
Pres513_6530	YpfJ protein, zinc metalloprotease superfamily	>2	NA
Pres513_6546	RNA polymerase ECF-type sigma factor	>2	GIP
**Pres513_6939**	Aldehyde dehydrogenase (EC 1.2.1.3)	ON	AM
Pres513_7181	Acetyl-CoA acetyltransferase (EC 2.3.1.9) @ 3-oxoadipyl-CoA thiolase (EC 2.3.1.174)	ON	BSM, MMDE, XDM, MTP, AM, LM
Pres513_7200	Quinohemoprotein amine dehydrogenase beta subunit (EC 1.4.99.-)	>2	NA

Referencias Fold Change: OFF (only without Mn); ON (only with Mn)

Kegg Pathway name

**BSM**	Biosynthesis of secondary metabolites	**GIP**	Genetic Information Processing
**MMDE**	Microbial metabolism in diverse environments	**T**	Transport
**CM**	Carbohydrate metabolism	**ST**	Signal Traduction
**LM**	Lipid metabolism	**B**	Biofilm
**BAM**	Biosynthesis of amino acids	**MCV**	Metabolism of cofactors and vitamins
**DCA**	Degradation of aromatic compound	**SS**	Secretion system
**EM**	Energy metabolism	**BMP**	Bacterial motility proteins
**NM**	Nuecleotide metabolism	**E**	Exosome
**AM**	Amino acid metabolism	**P**	Peroxisome
**MTP**	Metabolism of terpenoids and polyketides	**QS**	Quorum sensing
**XDM**	Xenobiotics biodegradation and metabolism	**AR**	Antimicrobial resistance
		**NA**	Not Assigned
