# Supplementary material for: Cyclic di-GMP Signaling Links Biofilm Formation and Mn(II) Oxidation in Pseudomonas resinovorans
Source: mBio. 2022 Nov 14;13(6):e02734-22. doi: 10.1128/mbio.02734-22 (PMC9765421; doi:10.1128/mbio.02734-22)
Supplement: TABLE S2 [file mbio.02734-22-s0007.pdf]

Table S2. KEGG pathway analysis of cluster 1 and cluster 2 proteins in Figure S6.

Down-regulated in MOB-513-p*dgcB* Cluster 1				
**Locus Tag**	**Protein**	**Kegg Pathway name**	**Student's T-test Significant MOB-513-p*dgcB* /MOB-513-pEmpty + Mn(II)**	**Student's T-test Significant MOB-513-p*dgcB* /MOB-513-pEmpty - Mn(II)**
Pres513_203	Probable transmembrane protein	NA		+
Pres513_322	Twitching motility protein PilT	BMP, SS	+	
Pres513_383	Gamma-glutamyl-putrescine oxidase (EC1.4.3.-)	AM	+	
Pres513_444	LuxR-type transcription regulator	GIP	+	
Pres513_518	Putative stomatin/prohibitin-family membrane protease subunit aq_911	NA	+	
Pres513_569	Precorrin-2 oxidase (EC 1.3.1.76) @ Sirohydrochlorin ferrochelatase activity of CysG (EC 4.99.1.4) / Uroporphyrinogen-III methyltransferase (EC 2.1.1.107)	BSM, MMDE, MCV	+	
Pres513_767	TldE protein, part of TldE/TldD proteolytic complex	NA	+	+
Pres513_791	Sulfate adenylyltransferase subunit 2 (EC 2.7.7.4)	BSM, MMDE, EM, NM, AM		+
Pres513_815	16S rRNA (cytosine(1402)-N(4))-methyltransferase (EC 2.1.1.199)	GIP	+	
Pres513_964	Pca regulon regulatory protein PcaR	GIP	+	
Pres513_1092	hypothetical protein	NA	+	
Pres513_1111	4-aminobutyraldehyde dehydrogenase (EC 1.2.1.19)	MMDE, AM	+	
Pres513_1728	Transcriptional regulator MexT	AR, GIP	+	+
Pres513_1729	Alcohol dehydrogenase, zinc-containing	NA	+	+
Pres513_1730	Transcriptional regulator, AraC family	GIP	+	
Pres513_1731	Isoquinoline 1-oxidoreductase alpha subunit (EC 1.3.99.16)	NA	+	+
Pres513_1732	Isoquinoline 1-oxidoreductase beta subunit (EC 1.3.99.16)	NA	+	+
Pres513_1864	Dienelactone hydrolase and related enzymes	NA		+
Pres513_1866	Dienelactone hydrolase and related enzymes	NA	+	
Pres513_2110	Transcriptional regulator, GntR family	GIP	+	
Pres513_2330	Lysophospholipase (EC 3.1.1.5); Monoglyceride lipase (EC 3.1.1.23)	NA		+
Pres513_2343	Ferric iron ABC transporter, iron-binding protein	T	+	
Pres513_2402	Succinylornithine transaminase (EC 2.6.1.81)	AM	+	
Pres513_2867	Dihydroorotase (EC 3.5.2.3)	NM		+
Pres513_2906	Dihydrolipoamide acyltransferase component of branched-chain alpha-keto acid dehydrogenase complex (EC 2.3.1.168)	BSM, MMDE, CM	+	+
Pres513_2908	Branched-chain alpha-keto acid dehydrogenase, E1 component, alpha subunit (EC 1.2.4.4)	BSM, MMDE, CM, ST		+
Pres513_2975	Beta-ketoadipate enol-lactone hydrolase (EC 3.1.1.24)	MMDE, DCA, XDM		+
Pres513_2989	Selenide,water dikinase (EC 2.7.9.3)	AM, GIP		+
Pres513_3061	CCA tRNA nucleotidyltransferase (EC 2.7.7.72)	GIP	+	
Pres513_3063	FIG004684: SpoVR-like protein	NA	+	
Pres513_3069	SSU rRNA (adenine(1518)-N(6)/adenine(1519)-N(6))-dimethyltransferase (EC 2.1.1.182)	GIP	+	
Pres513_3087	Anthranilate phosphoribosyltransferase (EC 2.4.2.18)	BSM, BAM, AM		+
Pres513_3089	Cyclic AMP receptor protein	ST, QS, B, GIP	+	
Pres513_3097	Dioxygenases related to 2-nitropropane dioxygenase	EM		+
Pres513_3338	hypothetical protein	BSM, MMDE, CM	+	
Pres513_3720	NAD-specific glutamate dehydrogenase (EC 1.4.1.2), large form	MMDE, EM, AM	+	
Pres513_3721	MoxR-like ATPase in aerotolerance operon	NA		+
Pres513_3731	4-aminobutyraldehyde dehydrogenase (EC 1.2.1.19)	MMDE, AM	+	
Pres513_3758	hypothetical protein	NA		+
Pres513_3886	Topoisomerase IV subunit B (EC 5.99.1.-)	GIP	+	
Pres513_4394	ATP-dependent protease subunit HslV (EC 3.4.25.2)	NA	+	
Pres513_4487	LOG family protein YgdH	NM	+	
Pres513_4641	Broad-substrate range phospholipase C (EC 3.1.4.3)	NA	+	
Pres513_4914	Uncharacterized glutathione S-transferase-like protein	AM, XDM, T	+	
Pres513_5188	Glutathione S-transferase, unnamed subgroup (EC 2.5.1.18)	AM, XDM, T	+	
Pres513_5297	Transcriptional regulator, AcrR family	NA		+
Pres513_5400	Cytochrome c oxidase polypeptide II (EC 1.9.3.1)	EM	+	
Pres513_5498	Putrescine transport ATP-binding protein PotG (TC 3.A.1.11.2)	T	+	
Pres513_5619	Outer membrane low permeability porin, OprD family => OccD6/OprQ involved in adhesion		+	+
Pres513_5736	Porphobilinogen synthase (EC 4.2.1.24)	BSM, MMDE, MCV, E		+
Pres513_5738	Exopolyphosphatase (EC 3.6.1.11)	NM	+	
Pres513_5763	Protein QmcA (possibly involved in integral membrane quality control)	NA	+	
Pres513_5957	Protein-L-isoaspartate O-methyltransferase (EC 2.1.1.77)	NA		+
Pres513_5975	3-hydroxyacyl-[acyl-carrier-protein] dehydratase, FabZ form (EC 4.2.1.59)	LM, MCV		+
Pres513_5985	Translation elongation factor Ts	GIP		+
Pres513_6341	Type IV fimbrial assembly, ATPase PilB	BMP, SS	+	
Pres513_6366	Transcriptional regulator, AsnC family	GIP	+	
Pres513_6439	GTP-binding protein Obg	GIP	+	
Pres513_6505	Phenylalanine-4-hydroxylase (EC 1.14.16.1)	BAM, AM, MCV	+	
Pres513_6615	Ubiquinone biosynthesis regulatory protein kinase UbiB	NA	+	
Pres513_6833	Inositol-1-monophosphatase (EC 3.1.3.25)	BSM, CM, ST	+	
Pres513_6884	Chemotaxis response - phosphatase CheZ	BMP		+

Up-regulated in MOB-513-p*dgcB* Cluster 2				
**Locus Tag**	**Protein**	**Kegg Pathway name**	**Student's T-test Significant MOB-513-p*dgcB* /MOB-513-pEmpty +Mn(II)**	**Student's T-test Significant MOB-513-p*dgcB* /MOB-513-pEmpty - Mn(II)**
Pres513_234	DNA topoisomerase I (EC 5.99.1.2)	GIP	+	
Pres513_311	hypothetical protein	NA	+	
Pres513_338	FIG002283: Isochorismatase family protein	NA	+	+
Pres513_430	Heat shock protein 60 family co-chaperone GroES	GIP	+	+
Pres513_498	hypothetical protein	NA		+
Pres513_523	hypothetical protein	NA	+	
Pres513_575	tRNA 5-methylaminomethyl-2-thiouridine synthase subunit TusD	GIP	+	
Pres513_639	Putative two-component sensor	ST, B	+	
Pres513_952	hypothetical protein	NA	+	
Pres513_1389	Acetyl-CoA acetyltransferase (EC 2.3.1.9)	BSM, MMDE, LM, CM, EM, AM, MTP, XDM, ST, E	+	+
Pres513_1410	Exoenzymes regulatory protein AepA precursor	NA	+	
Pres513_2273	hypothetical protein	NA	+	
Pres513_2385	Indolepyruvate ferredoxin oxidoreductase, alpha and beta subunits	NA		+
Pres513_2444	Carbamate kinase (EC 2.7.2.2)	MMDE, EM, NM, AM	+	
Pres513_2459	Putative polysaccharide export protein YccZ precursor	ST, B, T		+
Pres513_2487	Capsular polysaccharide synthesis enzyme CpsC, polysaccharide export	B, T	+	
Pres513_2800	Transcriptional regulator, GntR family	GIP	+	
Pres513_2941	FKBP-type peptidyl-prolyl cis-trans isomerase SlyD (EC 5.2.1.8)	GIP	+	
Pres513_2985	RNA polymerase associated protein RapA	GIP	+	
Pres513_3056	SSU ribosomel protein S21p	ribosome, GIP		+
Pres513_3339	hypothetical protein	NA	+	+
Pres513_3410	Putative outer membrane protein	T		+
Pres513_3455	Excinuclease ABC subunit A	GIP	+	
Pres513_3470	SSU ribosomel protein S14p (S29e) @ SSU ribosomel protein S14p (S29e), zinc-independent	ribosome, GIP	+	+
Pres513_3475	LSU ribosomel protein L29p (L35e)	ribosome, GIP	+	
Pres513_3494	LSU ribosomel protein L11p (L12e)	ribosome, GIP		+
Pres513_3498	hypothetical protein	NA		+
Pres513_3786	LSU ribosomel protein L33p @ LSU ribosomel protein L33p, zinc-independent	ribosome, GIP	+	
Pres513_3959	hypothetical protein	NA		+
Pres513_4016	Biotin carboxyl carrier protein of acetyl-CoA carboxylase	BSM, MMDE, LM, CM, EM	+	
Pres513_4032	Urea ABC transporter, substrate binding protein UrtA	T	+	+
Pres513_4082	SSU ribosomel protein S18p @ SSU ribosomel protein S18p, zinc-independent	ribosome, GIP	+	
Pres513_4617	Transcription elongation factor GreA	GIP	+	
Pres513_4618	Carbamoyl-phosphate synthase large chain (EC 6.3.5.5)	NM, AM	+	
Pres513_4652	Cold shock protein of CSP family	NA	+	
Pres513_5067	Transcriptional repressor of PutA and PutP / Proline dehydrogenase (EC 1.5.5.2) / Delta-1-pyrroline-5-carboxylate dehydrogenase (EC 1.2.1.88)	BSM, AM, GIP		+
Pres513_5136	Nitrogen regulation protein NR(I), GlnG (=NtrC)	ST	+	
Pres513_5139	FIG00956533: hypothetical protein	NA		+
Pres513_5142	Glutamine synthetase type I (EC 6.3.1.2)	MMDE, BAM, CM, EM, AM, ST, E	+	
Pres513_5308	Copper metallochaperone, bacterial analog of Cox17 protein	GIP	+	
Pres513_5398	hypothetical protein	NA	+	
Pres513_5567	twitching motility protein PilH	ST, B, BMP, SS	+	
Pres513_5673	LSU ribosomel protein L32p @ LSU ribosomel protein L32p, zinc-independent	ribosome, GIP	+	
Pres513_5712	Acetate permease ActP (cation/acetate symporter)	T	+	
Pres513_5797	putative secreted protein	NA	+	
Pres513_6001	Cold shock protein of CSP family	NA	+	+
Pres513_6010	hypothetical protein	NA	+	
Pres513_6033	Helicase subunit of the DNA excision repair complex	NA	+	
Pres513_6065	Glutamate-ammonia-ligase adenylyltransferase (EC 2.7.7.42)	NA	+	
Pres513_6308	hypothetical protein	NA		+
Pres513_6320	Coenzyme PQQ synthesis protein D	NA		+
Pres513_6408	hypothetical protein	NA	+	
Pres513_6442	SSU ribosomel protein S20p	ribosome, GIP	+	+
Pres513_6445	Isoleucyl-tRNA synthetase (EC 6.1.1.5)	GIP	+	
Pres513_6698	Outer membrane protein W precursor	NA	+	+
Pres513_6745	Nitrate ABC transporter, substrate-binding protein	EM, T	+	
Pres513_6827	Iron-sulfur cluster assembly iron binding protein IscA	GIP	+	
Pres513_6840	Cold shock protein of CSP family	NA	+	+
Pres513_7037	Nonspecific porin and structural outer membrane protein OprF	T	+	+
Pres513_7081	UPF0145 protein YbjQ	NA	+	+

Kegg Pathway Name

**BSM**	Biosynthesis of secondary metabolites	**GIP**	Genetic Information Processing
**MMDE**	Microbial metabolism in diverse environments	**T**	Transport
**CM**	Carbohydrate metabolism	**ST**	Signal Traduction
**LM**	Lipid metabolism	**B**	Biofilm
**BAM**	Biosynthesis of amino acids	**MCV**	Metabolism of cofactors and vitamins
**DCA**	Degradation of aromatic compound	**SS**	Secretion system
**EM**	Energy metabolism	**BMP**	Bacterial motility proteins
**NM**	Nuecleotide metabolism	**E**	Exosome
**AM**	Amino acid metabolism	**QS**	Quorum sensing
**MTP**	Metabolism of terpenoids and polyketides	**AR**	Antimicrobial resistance
**XDM**	Xenobiotics biodegradation and metabolism	NA	Not assigned
