# Supplementary material for: Cyclic di-GMP Signaling Links Biofilm Formation and Mn(II) Oxidation in Pseudomonas resinovorans
Source: mBio. 2022 Nov 14;13(6):e02734-22. doi: 10.1128/mbio.02734-22 (PMC9765421; doi:10.1128/mbio.02734-22)
Supplement: TABLE S3 [file mbio.02734-22-s0008.pdf]

Table S3. KEGG pathway analysis of differentially expressed ON proteins in MOB-513-p*dgcB* in the absence (-Mn) or the presence (+Mn) of manganese.

Differentially expressed proteins MOB-513-p*dgcB* /MOB-513-pEmpty - Mn(II)			
**Locus Tag**	**Protein**	ON	**Kegg Pathway name**
fig|286.1388.peg.1099	Signal transduction histidine kinase CheA	MOB-513-pEmpty	B, ST
fig|286.1388.peg.1617	Pyruvate dehydrogenase E1 component (EC 1.2.4.1)	MOB-513-pEmpty	CM, BSM, MMDE
fig|286.1388.peg.1725	Multidrug efflux system, outer membrane factor lipoprotein => OprN of MexEF-OprN system	MOB-513-pEmpty	T
fig|286.1388.peg.1730	Transcriptional regulator, AraC family	MOB-513-pEmpty	GIP
fig|286.1388.peg.2130	D-amino acid dehydrogenase (EC 1.4.99.6)	MOB-513-pEmpty	AM
fig|286.1388.peg.2619	Transcriptional regulator, AraC family	MOB-513-pEmpty	GIP
fig|286.1388.peg.3277	Spermidine/putrescine import ABC transporter substrate-binding protein PotD (TC 3.A.1.11.1)	MOB-513-pEmpty	T
fig|286.1388.peg.3505	Glycolate dehydrogenase (EC 1.1.99.14), FAD-binding subunit GlcE	MOB-513-pEmpty	CM, BSM, MMDE
fig|286.1388.peg.3637	Exodeoxyribonuclease V beta chain (EC 3.1.11.5)	MOB-513-pEmpty	GIP
fig|286.1388.peg.5226	Low molecular weight protein tyrosine phosphatase (EC 3.1.3.48)	MOB-513-pEmpty	ST
fig|286.1388.peg.5632	Similar to citrate lyase beta chain, 3	MOB-513-pEmpty	NA
fig|286.1388.peg.11	DNA-binding response regulator KdpE	MOB-513-pBdgc	QS
fig|286.1388.peg.873	Nitrate ABC transporter, substrate-binding protein	MOB-513-pBdgc	T
fig|286.1388.peg.2698	TonB-dependent ferric achromobactin receptor protein	MOB-513-pBdgc	T
fig|286.1388.peg.3549	hypothetical protein	MOB-513-pBdgc	NA
fig|286.1388.peg.4417	Universal stress protein UspA and related nucleotide-binding proteins	MOB-513-pBdgc	NA
fig|286.1388.peg.4659	serine/threonine protein kinase	MOB-513-pBdgc	NA
fig|286.1388.peg.5040	Transcriptional regulator, GntR family	MOB-513-pBdgc	GIP
fig|286.1388.peg.6854	Exonuclease SbcD	MOB-513-pBdgc	NA
fig|286.1388.peg.7013	RTX toxins and related Ca2+-binding proteins	MOB-513-pBdgc	NA
fig|286.1388.peg.7014	RTX toxins and related Ca2+-binding proteins	MOB-513-pBdgc	NA

Differentially expressed proteins MOB-513-p*dgcB* /MOB-513-Empty + Mn(II)			
**Locus Tag**	**Protein**	ON	**Kegg Pathway name**
fig|286.1388.peg.11	DNA-binding response regulator KdpE	MOB-513-pEmpty	QS, ST
fig|286.1388.peg.71	CBS domain protein	MOB-513-pEmpty	NA
fig|286.1388.peg.775	Lipopolysaccharide export system protein LptC	MOB-513-pEmpty	NA
fig|286.1388.peg.1186	hypothetical protein	MOB-513-pEmpty	NA
fig|286.1388.peg.1676	Multidrug efflux system MdtABC-TolC, membrane fusion component MdtA	MOB-513-pEmpty	ST
fig|286.1388.peg.1719	Glutathione S-transferase (EC 2.5.1.18)	MOB-513-pEmpty	NA
fig|286.1388.peg.1725	Multidrug efflux system, outer membrane factor lipoprotein => OprN of MexEF-OprN system	MOB-513-pEmpty	T
fig|286.1388.peg.1726	Multidrug efflux system, inner membrane proton/drug antiporter (RND type) => MexF of MexEF-	MOB-513-pEmpty	T
fig|286.1388.peg.1727	Multidrug efflux system, membrane fusion component => MexE of MexEF-OprN system	MOB-513-pEmpty	T
fig|286.1388.peg.1800	Cyanoalanine nitrilase (EC 3.5.5.4)	MOB-513-pEmpty	XBM, AM, EM, MMDE
fig|286.1388.peg.2118	3-oxoacyl-[acyl-carrier protein] reductase (EC 1.1.1.100)	MOB-513-pEmpty	AM, NM
fig|286.1388.peg.2130	D-amino acid dehydrogenase (EC 1.4.99.6)	MOB-513-pEmpty	AM
fig|286.1388.peg.2362	Transcriptional regulator, LysR family	MOB-513-pEmpty	GIP
fig|286.1388.peg.2552	Transcriptional regulator, AraC family	MOB-513-pEmpty	GIP
fig|286.1388.peg.2969	Methyl-accepting chemotaxis sensor/transducer protein	MOB-513-pEmpty	BC, ST
fig|286.1388.peg.3230	YihE protein, required for LPS synthesis	MOB-513-pEmpty	NA
fig|286.1388.peg.3903	Cation transport ATPase	MOB-513-pEmpty	T
fig|286.1388.peg.4541	GGDEF domain/EAL domain protein	MOB-513-pEmpty	NA
fig|286.1388.peg.4658	Protein phosphatase 2C-like	MOB-513-pEmpty	NA
fig|286.1388.peg.5377	DOPA 4,5-dioxygenase (EC 1.14.99.-)	MOB-513-pEmpty	NA
fig|286.1388.peg.6972	Cytochrome c heme lyase subunit CcmF	MOB-513-pEmpty	NA
fig|286.1388.peg.7063	3,4-dihydroxy-2-butanone 4-phosphate synthase (EC 4.1.99.12) / GTP cyclohydrolase II (EC 3.5.4.	MOB-513-pEmpty	MCV, BSM, BoC
fig|286.1388.peg.7194	Uncharacterized amino acid permease, GabP family	MOB-513-pEmpty	NA
fig|286.1388.peg.141	Catalase KatE (EC 1.11.1.6)	MOB-513-pBdgc	ST, AM, CM, BSM
fig|286.1388.peg.320	hypothetical protein	MOB-513-pBdgc	P
fig|286.1388.peg.873	Nitrate ABC transporter, substrate-binding protein	MOB-513-pBdgc	T, EM
fig|286.1388.peg.4774	hypothetical protein	MOB-513-pBdgc	NA
fig|286.1388.peg.7013	RTX toxins and related Ca2+-binding proteins	MOB-513-pBdgc	NA
fig|286.1388.peg.7014	RTX toxins and related Ca2+-binding proteins	MOB-513-pBdgc	NA

Kegg Pathway Name

**BC**	Bacterial chemotaxis	**MCV**	Metabolism of cofactors and vitamins
**B**	Biofilm	**AM**	Amino acid metabolism
**QS**	Quorum sensing	**NM**	Nucleotide metabolism
**P**	Peroxisome	**EM**	Energy metabolism
**T**	transporters	**CM**	Carbohydrate metabolism
**ST**	Signal transduction	**BSM**	Biosynthesis of secondary metabolites
**GIP**	Genetic Information Processing	**MMDE**	Microbial metabolism in diverse environments
**XBM**	Xenobiotics biodegradation and metabolism	**BoC**	Biosynthesis of cofactors
		NA	Not Assigned
